# Pharmacologic improvement of CFTR function rapidly decreases sputum pathogen density, but lung infections generally persist

**DOI:** 10.1172/JCI167957

**Published:** 2023-05-15

**Authors:** David P. Nichols, Sarah J. Morgan, Michelle Skalland, Anh T. Vo, Jill M. Van Dalfsen, Sachinkumar B.P. Singh, Wendy Ni, Lucas R. Hoffman, Kailee McGeer, Sonya L. Heltshe, John P. Clancy, Steven M. Rowe, Peter Jorth, Pradeep K. Singh, the PROMISE-Micro Study Group

**Affiliations:** 1 Department of Pediatrics and; 2 Departments of Microbiology and Medicine, University of Washington, Seattle, Washington, USA.; 3 Therapeutics Development Network Coordinating Center, Seattle Children’s Research Institute, Seattle, Washington, USA.; 4 Department of Medicine, University of Iowa, Iowa City, Iowa, USA.; 5 Department of Medicine, University of Alabama, Birmingham, Alabama, USA.; 6 Departments of Pathology and Laboratory Medicine, Medicine, and Biomedical Sciences, Cedars-Sinai Medical Center, Los Angeles, California, USA.; 7 The PROMISE-Micro Study Group is detailed in Supplemental Acknowledgments.

**Keywords:** Microbiology, Pulmonology, Bacterial infections

## Abstract

**Background:**

Lung infections are among the most consequential manifestations of cystic fibrosis (CF) and are associated with reduced lung function and shortened survival. Drugs called CF transmembrane conductance regulator (CFTR) modulators improve activity of dysfunctional CFTR channels, which is the physiological defect causing CF. However, it is unclear how improved CFTR activity affects CF lung infections.

**Methods:**

We performed a prospective, multicenter, observational study to measure the effect of the newest and most effective CFTR modulator, elexacaftor/tezacaftor/ivacaftor (ETI), on CF lung infections. We studied sputum from 236 people with CF during their first 6 months of ETI using bacterial cultures, PCR, and sequencing.

**Results:**

Mean sputum densities of *Staphylococcus aureus*, *Pseudomonas aeruginosa*, *Stenotrophomonas maltophilia*, *Achromobacter* spp., and *Burkholderia* spp. decreased by 2–3 log_10_ CFU/mL after 1 month of ETI. However, most participants remained culture positive for the pathogens cultured from their sputum before starting ETI. In those becoming culture negative after ETI, the pathogens present before treatment were often still detectable by PCR months after sputum converted to culture negative. Sequence-based analyses confirmed large reductions in CF pathogen genera, but other bacteria detected in sputum were largely unchanged. ETI treatment increased average sputum bacterial diversity and produced consistent shifts in sputum bacterial composition. However, these changes were caused by ETI-mediated decreases in CF pathogen abundance rather than changes in other bacteria.

**Conclusions:**

Treatment with the most effective CFTR modulator currently available produced large and rapid reductions in traditional CF pathogens in sputum, but most participants remain infected with the pathogens present before modulator treatment.

**Trial Registration:**

ClinicalTrials.gov NCT04038047.

**Funding:**

The Cystic Fibrosis Foundation and the NIH.

## Introduction

Small-molecule drugs known as cystic fibrosis (CF) transmembrane conductance regulator (CFTR) modulators have transformed the care of most people with the genetic disease CF (1–6). These medications improve defective anion conductance of the CFTR channel in those with responsive CFTR variants, which is the physiological basis of CF. They also have pleiotropic therapeutic effects in this multiorgan-system disease. The most effective modulators improve lung function and respiratory symptoms, reduce flares of lung disease (i.e., pulmonary exacerbations), and increase body weight (5, 6). In addition, observational studies of people treated with modulators have increased our understanding of disease pathophysiology by linking increased CFTR activity to improvements in mucociliary clearance (7, 8), pH in the airway (9) and small intestine (5), smooth muscle tone (10), and immune cell function (11), among other effects.

The recently developed 3-drug combination modulator elexacaftor/tezacaftor/ivacaftor (ETI) is perhaps the most important advance in CF care to date (1–4). Laboratory and clinical studies have found that ETI has robust activity on the most common CFTR mutation (F508del). Thus, it is effective in people with 1 or 2 F508del alleles, which includes nearly 90% of people with CF (3, 4). Moreover, ETI produces the largest improvements in clinical outcomes among modulators approved for human use (1, 2).

While modulators produce consistent improvements in CFTR activity, lung function, and nutritional status, their effects on CF lung infections remain unclear. Some studies find desirable changes in airway infection after starting modulators. For example, US CF patient registry data demonstrated that treatment with the first modulator approved for humans (ivacaftor) was associated with decreased prevalence of sputum cultures positive for *Pseudomonas aeruginosa* (12–14). Small studies also found that ivacaftor rapidly decreased the sputum density of *P*. *aeruginosa* and *Staphylococcus aureus* and reduced lung inflammatory markers (15, 16). However, other studies have shown minimal changes in sputum bacteria after modulators (17, 18). Inconsistent findings across studies may be due to methodological differences, variations in participant characteristics, and limitations inherent to small study sizes.

Understanding how modulators affect CF airway infections is critical for a number of reasons. First, bacterial lung infection is a cardinal manifestation of CF and is thought to drive lung function decline and shorten survival (19, 20). Therefore, microbiological outcomes are important for defining the nature of residual disease after CFTR function has been markedly but incompletely restored. Second, understanding postmodulator lung infection will help direct future research and clinical care. For example, if pathogens frequently persist and infection remains linked to poor outcomes, then novel antimicrobial strategies may be needed to maximize health. Third, this work could help delineate poorly understood aspects of infection biology. CF airway infections, like many other chronic infections, are a consequence of host defense defects. Little is known about how infections respond when infection-initiating host defects (e.g., impaired mucociliary clearance) are significantly corrected, but secondary manifestations such as structural airway damage or pathogen adaptations already exist.

Here we investigated how the most effective CFTR modulator available (ETI) affected sputum microbiology in over 200 people with CF. This preplanned analysis of the PROMISE study (21) measured changes through the first 6 months of treatment by clinical prescription in the United States. We used both culture- and DNA-based methods to measure traditional CF pathogens and bacteria not traditionally considered CF pathogens to define modulator-induced changes in the bacterial composition of sputum.

## Results

### Study population and procedures.

PROMISE (ClinicalTrials.gov NCT04038047) (2) is a prospective, multicenter, observational study of the effects of ETI in people with CF. Data and specimens were collected before (baseline) and 1, 3, and 6 months after initiating treatment; visits continued through at least 30 months of ETI use. The microbiological study of PROMISE analyzed sputum samples from 236 participants collected between October 2019 and April 2021 (21). Microbiological study participants had an average age of 24.8 years (SD 10.9), were 52.5% female (*n* = 124), and 47.9% (*n* = 113) F508del *CFTR* homozygous. Additional demographic data and prior modulator use are reported in Table 1 and Supplemental Table 1; supplemental material available online with this article; https://doi.org/10.1172/JCI167957DS1 Participants were encouraged to continue baseline medications; however, use of inhaled antibiotics and hypertonic saline decreased significantly during ETI treatment (Supplemental Table 2).

The COVID-19 pandemic necessitated study modifications, as described previously (2). The baseline and 1-month visits were largely unaffected, but the 3- and 6-month visits occurred when in-person evaluation, sputum induction, and lung function testing were limited at some sites. Study visit completion was 83%, 39%, and 91% at 1, 3, and 6 months, respectively. One percent (*n* = 2), 2% (*n* = 2), and 3% (*n* = 6) of participants reported not using ETI at 1, 3, and 6 months, respectively. The study protocol favored sputum collection after saline inhalation (i.e., induced sputum collection), but spontaneously expectorated sputum was permitted and accounted for 13% (*n* = 27), 5% (*n* = 8), 7% (*n* = 5), and 57% (*n* = 66) of samples at baseline, 1, 3, and 6 months, respectively. The increase in spontaneously expectorated samples at later time points was due to restrictions on in-clinic sputum induction during the pandemic. Oropharyngeal swab samples were not allowed for this study.

### CFTR activity, lung function, body weight, and respiratory symptoms improve after ETI.

After 1 month of ETI, sweat chloride (which reflects CFTR activity) decreased by an average of 39.0 mmol/L (95% CI: –41.4, –36.6), lung function increased by 8.9% predicted forced expiratory volume in 1 second (FEV_1_pp) (95% CI: 7.8%, 10.0%), body mass index (BMI) increased by 0.41 kg/m^2^ among adults (95% CI: 0.29, 0.53), and respiratory symptoms improved by 10.5 Cystic Fibrosis Questionnaire – Revised (respiratory domain) (CFQ-R RD) score points (95% CI: 7.5, 13.4; minimum significant change is considered 4 points, ref. 22) (Table 2), consistent with prior studies (1–4). FEV_1_pp and sweat chloride were largely unchanged beyond 1 month of treatment, but BMI and respiratory symptom scores improved further at 6 months (mean BMI change from baseline in adults 1.12 kg/m^2^; mean CFQ-R RD score change +18.1 points) (Table 2).

**Figure 1:**
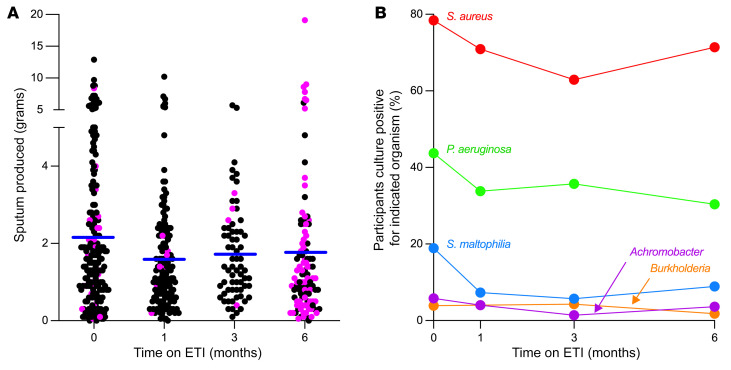
Sputum production and sputum culture positivity after ETI. (**A**) Grams of sputum produced after saline inhalation (black points) and spontaneously (pink points). Participants not producing sputum (due to inability or missed visit) are not shown. Blue lines indicate mean from all participants producing sputum at that visit. (**B**) Proportion of participants providing sputum samples that were culture positive for the indicated organism. Graph includes all participants, including those missing data at 1 or more study visit. Participants not providing sputum at a visit are not included in analysis of that visit. See Supplemental Figure 1 for complete case analyses. The number of participants studied at each time point is reported in Supplemental Table 3.

The proportion of participants attending study visits who produced sputum samples declined from 89% (*n* = 210/236) at baseline to 80% (*n* = 156/195), 77.2% (*n* = 71/92), and 53.5% (*n* = 115/215) at 1, 3, and 6 months, respectively (Supplemental Table 3). Thirty-two (15.9%) participants who produced sputum at baseline did not produce sputum at any of the posttreatment time points. The limited sputum collections at the 3- and 6-month visits are difficult to interpret due to pandemic-related modifications to study conduct at research sites. However, the decrease noted at the prepandemic 1-month visit likely reflects therapeutic effects of ETI. Average weight of sputum samples that were produced was 2.19 grams (range: 0.05–12.90) at baseline, and 1.62, 1.72, and 1.79 grams at 1, 3, and 6 months, respectively (Figure 1A).

### CF pathogen prevalence declined after ETI.

We examined the effects of ETI on lung infection by first measuring changes in the prevalence of sputum cultures positive for *S*. *aureus*, *P*. *aeruginosa*, *Stenotrophomonas maltophilia*, *Achromobacter* spp., and *Burkholderia* spp., which are traditionally considered CF pathogens (hereafter, “traditional CF pathogens”). Analysis of provided samples (i.e., including samples from participants who missed or were unproductive at 1 or more visits) showed that *P*. *aeruginosa* and *S*. *maltophilia* prevalence declined during the first month of ETI and were relatively stable thereafter (*P*. *aeruginosa* declined from 43.7% to 33.8% at 1 month, change of 9.9%, *P* = 0.0009; *S*. *maltophilia* declined from 18.9% to 7.3% at 1 month, change of 11.6%, *P* = 0.0009) (Figure 1B). *S*. *aureus* and *Achromobacter* and *Burkholderia* spp. prevalence was not significantly changed (Figure 1B). Complete case analyses (i.e., eliminating participants with missed visits or not producing sputum at visits) showed similar trends (Supplemental Figure 1).

### Pathogen density markedly declined after ETI, but most participants remained infected.

**Figure 2:**
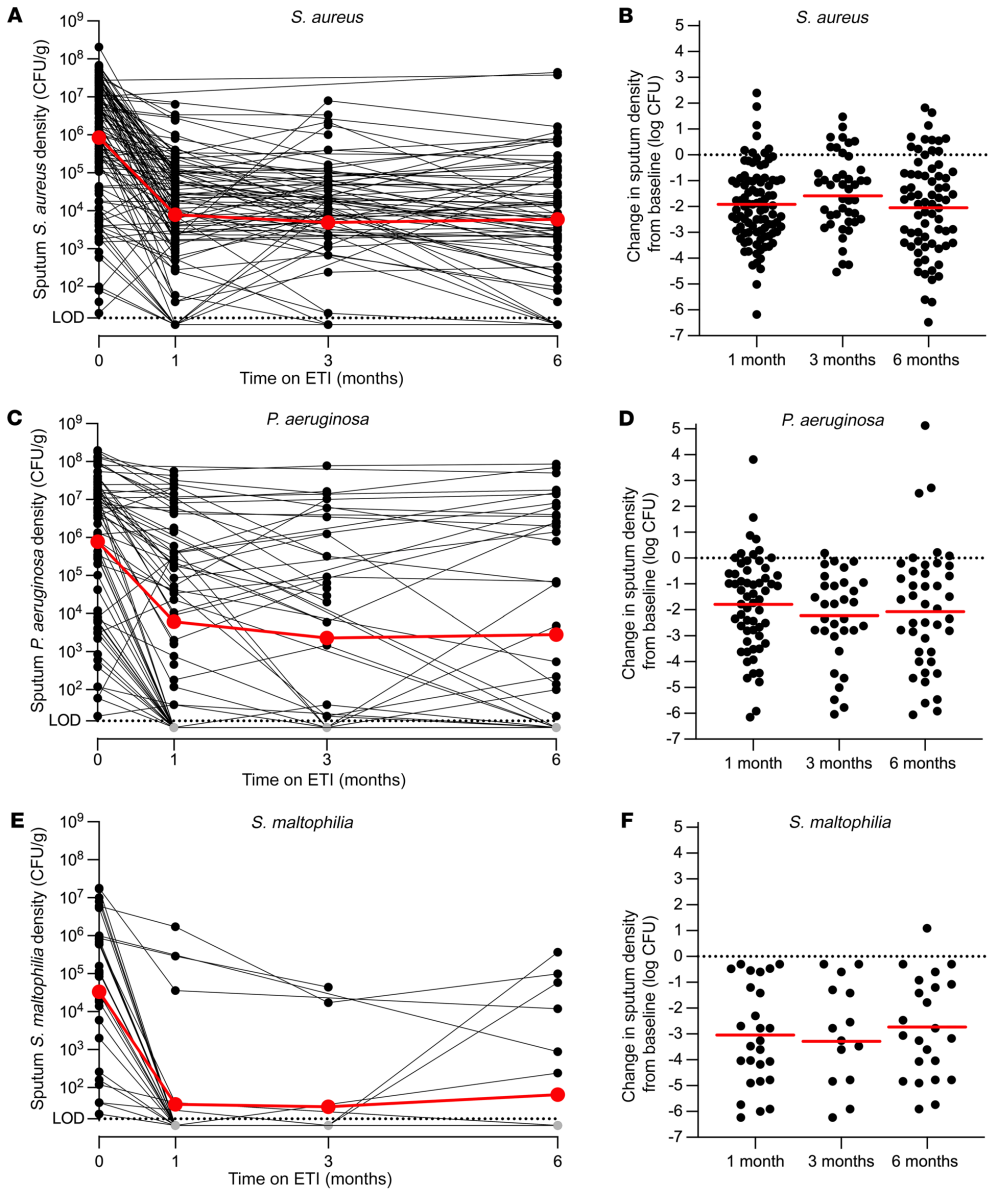
Sputum density of CF pathogens declines after ETI. (**A**, **C**, and **E**) Density of indicated pathogens measured by quantitative culture for participants who were culture positive for the indicated pathogen at the baseline visit. Data from individual participants are indicated in black, averages are indicated in red. The limit of detection was 20 CFU/mL (dotted line). (**B**, **D**, and **F**) By-participant changes in sputum density of indicated pathogens measured by quantitative culture for participants who were culture positive for the indicated pathogen at the baseline visit. Data from individual participants are indicated in black, averages are indicated in red. Changes from baseline to 1 month were significant by repeated measures analysis for each organism (*P* < 0.0001). See Supplemental Table 4 for average values, the number of participants studied per time point, and statistical analysis; and Supplemental Figure 2 for data on *Burkholderia* and *Achromobacter* spp.

We measured changes in the sputum culture density of pathogens in participants who were pathogen culture positive before treatment was initiated. One month after starting ETI, average sputum *S*. *aureus* density in the cohort declined by 2.03 log_10_ CFU/mL (95% CI: –2.28, –1.68; *n* = 94) (Figure 2A), *P*. *aeruginosa* declined by an average 2.11 log_10_ CFU/mL (95% CI: –2.21, –1.02; *n* = 60) (Figure 2C), and *S*. *maltophilia* declined by an average of 2.96 log_10_ CFU/mL (95% CI: –3.90, –2.32; *n* = 26) (Figure 2E). *Achromobacter* and *Burkholderia* spp. showed similar declines, although the numbers of participants entering the study culture positive for these organisms were small (Supplemental Figure 2). Average by-participant pathogen density changes were similar (Figure 2, B, D, and F, Supplemental Figure 2, and Supplemental Table 4). The average change in *P*. *aeruginosa* sputum density was approximately 10-fold smaller when only culture-positive samples were considered (Supplemental Figure 3), indicating that some of the change was attributable to samples that became at least transiently culture negative (see below).

Average culture densities of *S*. *aureus* and *P*. *aeruginosa* changed minimally after the first month of ETI (Figure 2 and Supplemental Table 4). These findings suggest that the marked initial declines in pathogen density were followed by a new steady state wherein pathogen growth and elimination become relatively balanced. Sputum pathogen density measurements using droplet digital PCR (ddPCR) (Supplemental Figure 4; see controls and validation in Supplemental Figure 5) and 16S rRNA gene sequencing (see below) produced similar results.

It was notable that a small number of participants entered the study culture negative for one of the measured pathogens and then became culture positive while on treatment (Supplemental Figure 6). In some cases, patient registry data identified no previous positive cultures for the detected organism in the previous 2 years (Supplemental Figure 6). This observation raises the possibility that some treated participants had residual lung host-defense abnormalities that conferred infection susceptibility. However, pathogen abundance was generally low in these newly identified infections (Supplemental Figure 6), and it remains unclear whether infection will be transient or of long duration.

### Most participants remained infected with the pathogens present before starting ETI.

Eradication of established CF infections is a sought-after clinical objective but is generally rare with existing treatments. However, CFTR modulators could be more effective, as they may improve multiple host defenses (7, 8, 11). We measured the proportion of participants who were sputum culture positive for each pathogen at baseline, and then became repeatedly culture negative after starting ETI. To be considered repeatedly culture negative, all sputum samples collected after ETI had to be culture negative for the pathogen present before treatment, with a minimum of 2 samples provided after initiating ETI.

**Figure 3:**
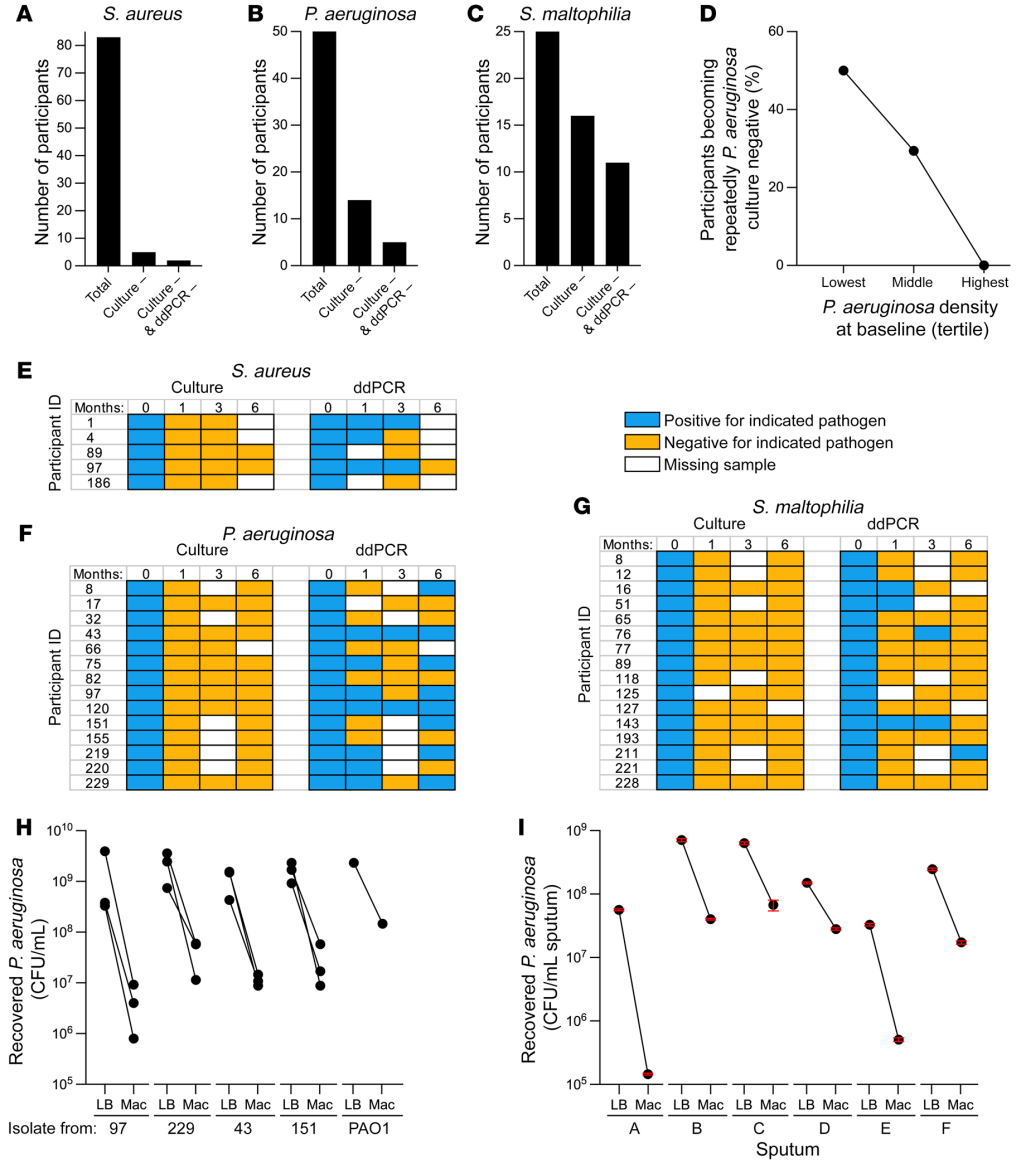
Some participants become repeatedly culture negative for CF pathogens after ETI. (**A**–**C**) The number of participants with sputum cultures positive for *Staphylococcus aureus* (**A**), *Pseudomonas aeruginosa* (**B**), or *Stenotrophomonas* *maltophilia* (**C**) at the baseline visit who provided at least 2 post-ETI samples are indicated as “Total”. The number of participants whose sputum became repeatedly culture negative (i.e., all samples after the baseline visit were culture negative) for the indicted pathogen are indicated as “Culture –”. The number of participants whose sputum became repeatedly culture and ddPCR negative (i.e., all samples after the baseline visit were culture and ddPCR negative) are indicated as “Culture – & ddPCR –”. (**D**) Inverse relationship between *P*. *aeruginosa* sputum culture density at the baseline visit and transition to repeatedly *P*. *aeruginosa* culture–negative status after ETI. Baseline *P*. *aeruginosa* culture density was categorized by tertile; the highest tertile exceeded 1.08 × 10^7^, middle was between 1.08 × 10^7^ and 4.21 × 10^4^, and lowest was less than or equal to 4.21 × 10^4^ *P*. *aeruginosa* CFU/gm of sputum. Significance of differences was significant by Fisher’s exact test, *P* = 0.005. See Supplemental Table 8 for the number of participants analyzed and statistical analysis. (**E**–**G**) ddPCR assays detect pathogens in some culture-negative sputum samples. Participants becoming repeatedly culture negative for *S*. *aureus* (**E**), *P*. *aeruginosa* (**F**), or *S*. *maltophilia* (**G**) are indicated in rows and results of pathogen detection by culture and ddPCR are indicated by study time point in columns. Positive culture or ddPCR results are indicated in blue; negative culture or ddPCR results are indicated in yellow; missing data are indicated by white. See Supplemental Figure 7 for results of ddPCR assays in participants culture positive for *P*. *aeruginosa* and *S*. *aureus*. (**H** and **I**) Selective media used for *P*. *aeruginosa* culture reduced recovery of *P*. *aeruginosa*. Three isolates each from 4 PROMISE participants and the reference strain PAO1 (**H**) grown to late stationary phase, and sputum from 6 people with CF (**I**) were cultured on nonselective LB agar (LB) or the selective MacConkey agar (Mac) used for sputum *P*. *aeruginosa* culture in this study and by clinical labs generally, and viable *P*. *aeruginosa* counts measured (see Methods). CFU recovered from LB was higher than CFU recovered from MacConkey (*P* < 0.05 for all samples) by multiple unpaired, 2-tailed *t* tests on log-transformed values.

Six percent (5/83) of participants entering the study with *S*. *aureus* became repeatedly culture negative, as did 28% (14/50) with *P*. *aeruginosa*, 64% (16/25) with *S*. *maltophilia* (Figure 3, A–C), 43% (3/7) with *Achromobacter* spp., and 25% (1/4) with *Burkholderia* spp. This analysis considers the first approximately 6 months of ETI use, and the proportion of participants becoming repeatedly culture negative may change with additional follow-up. Similar rates of conversion to repeatedly culture-negative status were observed in participants providing samples at every study visit (Supplemental Table 5), although fewer participants met this criterion.

We considered the possibility that the requirement for 2 or more culture-negative sputum samples might underestimate the proportion of participants becoming repeatedly culture negative, as culture-negative participants might fail to produce sputum at higher rates than participants with positive cultures. However, only approximately 9% of participants in the overall cohort were eliminated from this analysis due to failed sputum induction attempts (Supplemental Table 6). Moreover, a hypothetical analysis that considered failed sputum inductions as culture-negative cases did not appreciably increase the proportion of participants considered repeatedly culture negative (Supplemental Table 7).

We examined infection characteristics associated with conversion to repeatedly culture negative, focusing on *P*. *aeruginosa*, as this organism has been linked to lung function decline and is the greatest focus of eradication treatment in clinical practice (19, 23, 24). Participants that could be considered to have “chronic” *P*. *aeruginosa* infections (see Methods for definition) and participants with mucoid *P*. *aeruginosa* identified in study cultures became repeatedly culture negative at numerically lower rates than those who did not, but these differences were not statistically significant (Supplemental Table 8). However, there was a statistically significant inverse relationship between conversion to repeatedly culture-negative status and participants’ baseline sputum *P*. *aeruginosa* densities (Supplemental Table 8). We found that 50% of participants with the lowest tertile of baseline *P*. *aeruginosa* density, 29.4% of participants with the middle tertile, and none with the highest tertile converted to repeatedly *P*. *aeruginosa* culture negative (*P* = 0.005) (Figure 3D). We also examined associations between conversion to repeatedly culture-negative status and pre-ETI lung function (ppFEV_1_), inhaled antibiotic and hypertonic saline use, and participant age and found no significant associations (Supplemental Table 8). Future work involving longer follow-up and analysis of participant characteristics associated with infection status will be informative (see Discussion).

### ddPCR suggested continued pathogen presence in most culture-negative participants.

Given the clinical importance of infection clearance, we used species-specific ddPCR assays to detect the pathogen(s) cultured at baseline, in sputum that became repeatedly culture negative (when sputum was available). ddPCR fractionates samples into tens of thousands of droplets and performs PCR on each to enable rare target quantitation (25). ddPCR detection indicated the presence of at least 200 genome copies/mL (Supplemental Figure 5, D–F).

Culture and ddPCR assays were generally concordant in participants who were culture positive for *P*. *aeruginosa* and *S*. *aureus* (Supplemental Figure 7). However, in participants with repeatedly culture-negative results after ETI initiation, ddPCR detected the pathogen(s) cultured at baseline in at least 1 culture-negative sputum sample in 60% (3/5) of participants who were *S*. *aureus* positive at baseline, 64% (9/14) of participants *P*. *aeruginosa* positive at baseline, and 31% (5/16) of participants *S*. *maltophilia* positive at baseline (Figure 3, A–C and E–G).

Experiments using *P*. *aeruginosa* isolates (Figure 3H) and CF sputum (Figure 3I) suggest that the growth-inhibiting effects of selective media (used here and generally by clinical labs) may contribute to culture ddPCR discordance for *P*. *aeruginosa*. It is important to note that because contamination with oropharyngeal secretions is unavoidable in expectorated sputum, the ddPCR signal in sputum from participants who became repeatedly culture negative may not have originated from the lung (see Discussion). This explanation may be particularly relevant for *S*. *aureus*, which frequently colonizes the nasopharynx.

### Persistent ddPCR positivity likely indicates viable bacteria.

ddPCR positivity could have been due to residual DNA from killed bacteria. To investigate this possibility, we treated culture-negative, ddPCR-positive sputum samples with DNase to degrade free bacterial DNA (i.e., DNA from bacteria with permeabilized membranes), before we lysed bacterial cells for DNA purification. Control experiments showed that extracellular DNase reduced the signal from permeabilized bacteria by approximately 4 log_10_ genome copies/mL (Supplemental Figure 8). In 6 of 7 participants who became repeatedly *P*. *aeruginosa* culture negative and provided adequate sputum volume for these analyses, 1 or more of the culture-negative samples remained ddPCR positive for *P*. *aeruginosa* after DNase treatment (Supplemental Figure 9). The same was true for 2 of 2 participants who became repeatedly *S*. *aureus* culture negative and provided adequate sputum volume for these experiments (Supplemental Figure 9).

It was also notable that sputum from *S*. *aureus*– and *P*. *aeruginosa*–infected participants were ddPCR positive at an average of 83 days (range 45–103 days) and 328 days (range 33–319 days), respectively, after the last culture-positive sputum. The long duration of ddPCR positivity after sputum became culture negative along with the results from the DNase-treated samples suggest that viable *S*. *aureus* and *P*. *aeruginosa* cells are present in most participants who became repeatedly culture negative.

### Sequencing shows CF pathogens markedly decline, but other bacteria show minor changes.

Clinical cultures focus on identifying pathogens predicted to cause disease at the location of interest (e.g., sputum culture methods target traditional lung pathogens). In contrast, sequence-based (i.e., microbiome) methods measure the bacterial content of samples more comprehensively. We used DNA-based methods to measure the absolute abundances of genera found at an average relative abundance of 1% or greater in pre-ETI samples. Genera absolute abundances were calculated as the product of total bacterial abundance (measured by 16S rRNA gene ddPCR) and genera relative abundance (measured by 16S rRNA amplicon sequencing). Control experiments (ref. 26 and Supplemental Figures 10 and 11) show the validity of this approach.

**Figure 4:**
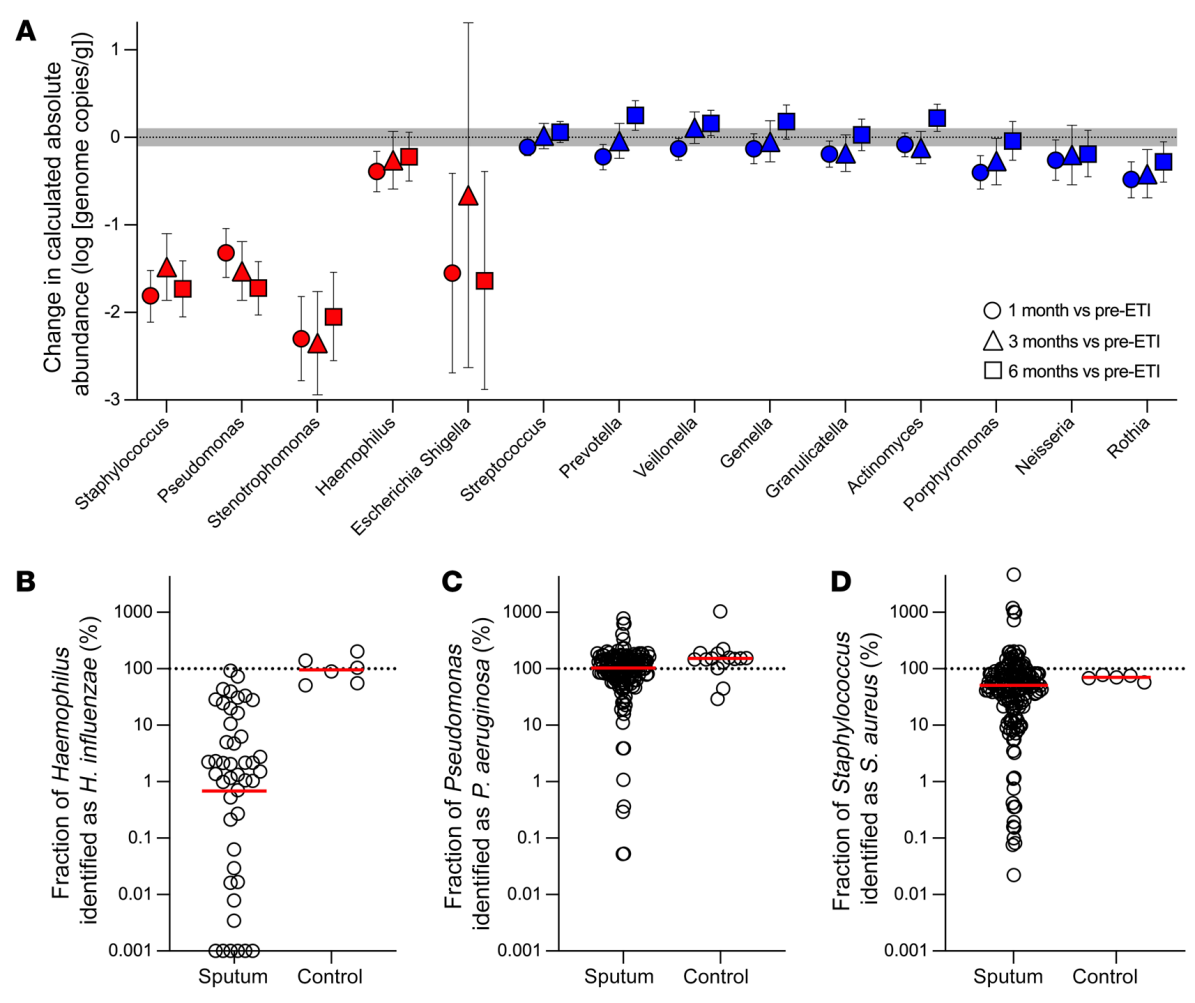
Sequence-based analyses find marked declines in the sputum density of traditional CF pathogens, but little change in other organisms. (**A**) By-participant change in calculated absolute abundance after 1 (circles), 3 (triangles), and 6 (squares) months of ETI for genera detected at an average of 1% or greater in baseline sputum samples. Genera containing a traditional CF pathogen are indicated with red symbols, other bacterial genera are indicated in blue symbols. Mean and CIs as determined by mixed-model repeated measures analysis are shown. Both groups are ordered by the average cohort-wide relative abundance at baseline (high to low). Gray shading indicates technical variation in measurements of control samples (see Supplemental Figure 10C). See Supplemental Table 9 for number participants studied per time point and statistical analysis. (**B**–**D**) Proportion of *Haemophilus* (**B**), *Pseudomonas* (**C**), and *Staphylococcus* (**D**) genera abundance attributable to the corresponding CF pathogen species. The proportion of *Haemophilus*, *Pseudomonas*, and *Staphylococcus* genera genomes that are *H*. *influenzae*, *P*. *aeruginosa*, or *S*. *aureus* species, respectively, was calculated by dividing *H*. *influenzae*, *P*. *aeruginosa*, or *S*. *aureus* species genome abundance measured by species-specific ddPCR by the corresponding genera absolute abundance. Sputum from participants with prior evidence of *H*. *influenzae*, *P*. *aeruginosa*, or *S*. *aureus* (from patient registry data) were studied. “Controls” were replicate cultures of laboratory strains of the indicated species. Values for controls sometimes exceed 100% due to variations in measurements of genera absolute abundance (using the product of total 16S rRNA and genera relative abundance) and species absolute abundance (using species specific ddPCR data). The difference between *Haemophilus* genera and *H*. *influenzae* abundance was significant (*P* = 0.0014); differences between *Pseudomonas* and *P*. *aeruginosa* abundance (*P* = 0.09) and *Staphylococcus* and *S*. *aureus* abundance (*P* = 0.38) were not different by 2-tailed *t* test on log-transformed data.

Of 14 genera present at an average relative abundance of 1% or greater in pre-ETI samples, only 4 decreased by 10-fold or more after ETI (Figure 4A). All 4 of these genera included species considered to be traditional CF pathogens, including *Staphylococcus*, *Pseudomonas*, *Stenotrophomonas*, and *Escherichia*/*Shigella* (average reduction of –1.75 log_10_ genome copies/mL at 1 month; range –2.3 to –1.32; Figure 4A and Supplemental Table 9). Consistent with the culture findings, most of the decline occurred in the first month of treatment (Figure 4A).

The differential response of *Haemophilus* (minimally changed after ETI) and other traditional CF pathogen genera (markedly decreased) led us to investigate the extent to which the *Haemophilus* genus-level reads reflected the species *H*. *influenzae* (the traditional CF pathogen in the genus) or other *Haemophilus* species, which can be abundant in the oropharynx (27). We performed *H*. *influenzae*–specific ddPCR on sputum from 16 participants previously known from patient registry data to be *H*. *influenzae* infected. Even in these participants known to be *H*. *influenzae* infected, *H*. *influenzae* accounted for an average of 0.68% (median 1.5%, range 0.001%–92%) of the *Haemophilus* genus sequence reads (Figure 4B). In contrast, most of the *Pseudomonas* and *Staphylococcus* genera signals were attributable to *P*. *aeruginosa* and *S*. *aureus* (Figure 4, C and D). Thus, the lack of change in the *Haemophilus* genus may be due to the fact that genera-based measurements predominantly represent *Haemophilus* species abundant in the oropharynx and not considered traditional CF pathogens (e.g., *H*. *parainfluenzae*, *H*. *segnis*, *H*. *aphrophilus*, and others) (27).

**Figure 5:**
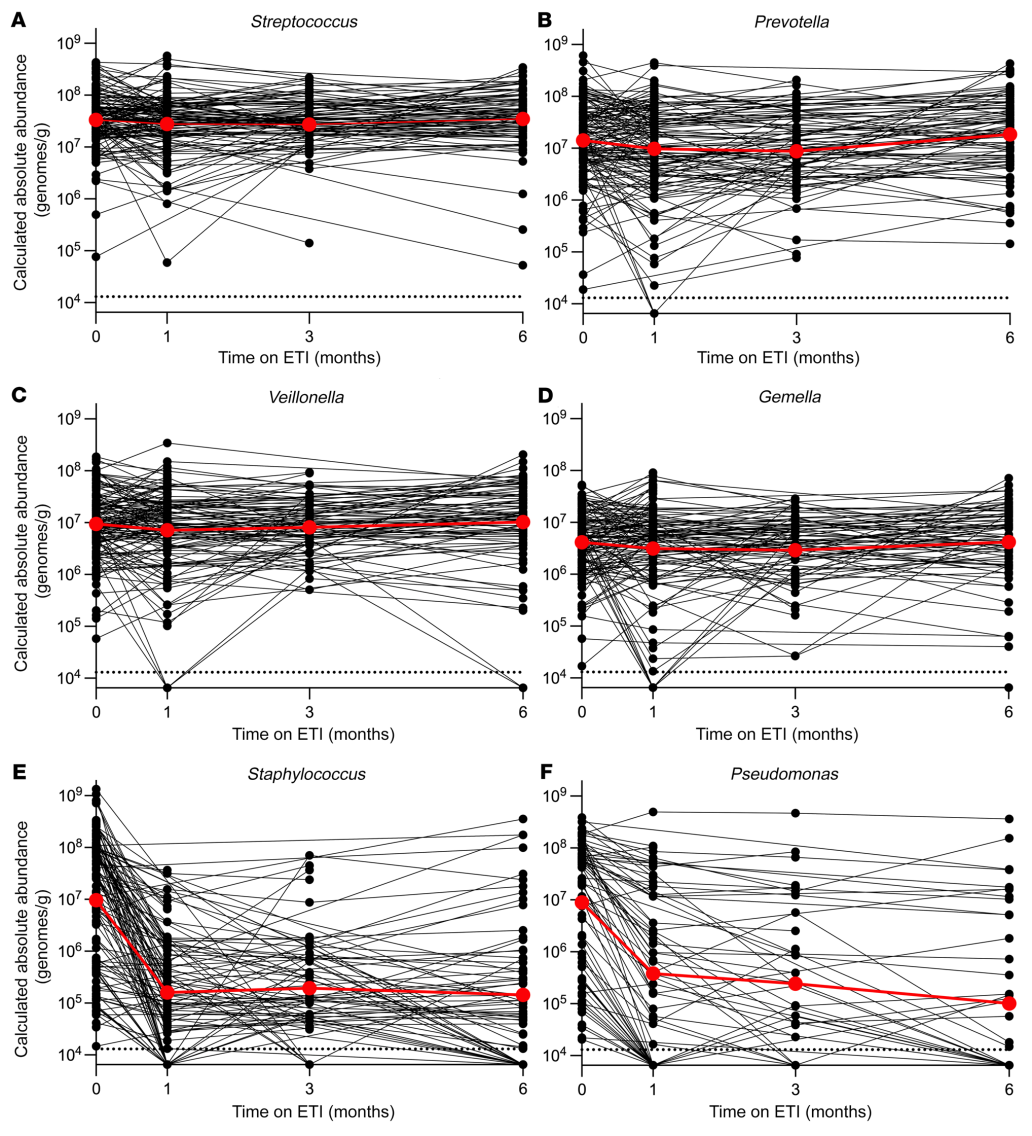
Sequence-based analyses show that other organisms in sputum exhibit little change, whereas traditional CF pathogens decrease. Change in calculated absolute abundance of *Streptococcus* (**A**), *Prevotella* (**B**), *Veillonella* (**C**), *Gemella* (**D**), *Staphylococcus* (**E**), and *Pseudomonas* (**F**) genera in participants’ sputum. Data from individual participants are indicated in black, averages are indicated in red. Participants for which the indicated genera was not detected in baseline sputum samples were not included. See Supplemental Table 9 for the number of samples analyzed per time point and statistical analysis.

We examined the differential responses of genera traditionally considered CF pathogens and other genera (called “other bacteria” below) to ETI in 2 additional ways. We compared absolute abundance changes in the 4 most abundant other bacterial genera detected in participants’ sputum individually (*Streptococcus*, *Prevotella*, *Veillonella*, and *Gemella*) (Figure 5, A–D) with changes in *Pseudomonas* and *Staphylococcus* (Figure 5, E–F). We also compared absolute abundance changes in other bacteria as a group (including those with <1% relative abundance in pre-ETI samples) with changes in CF pathogen genera as a group (Supplemental Figure 12, A and B). Consistent with findings above, these analyses showed that traditional CF pathogen genera markedly declined, whereas other bacteria showed little change.

Two additional points related to this analysis were notable. First, examining the same data using relative rather than absolute abundance measurements obscured the fact that other bacteria showed minor changes after ETI (Supplemental Figure 12, C and D). This is because decreases in CF pathogens will result in the appearance of reciprocal changes in other bacteria even when their absolute concentrations are unchanged. Second, total bacterial abundance measurements in sputum (measured using 16S rRNA gene ddPCR) were unchanged after ETI (Supplemental Figure 12E). The lack of change in total bacterial abundance indicates that other bacteria were generally more abundant in sputum than CF pathogens. Our data do not enable us to determine whether these other bacteria originate from the lung or are contributed by oropharyngeal secretions, which are known to contain a high density of these organisms (see Discussion).

### Changes in microbial diversity and composition after ETI are due to CF pathogen declines.

**Figure 6:**
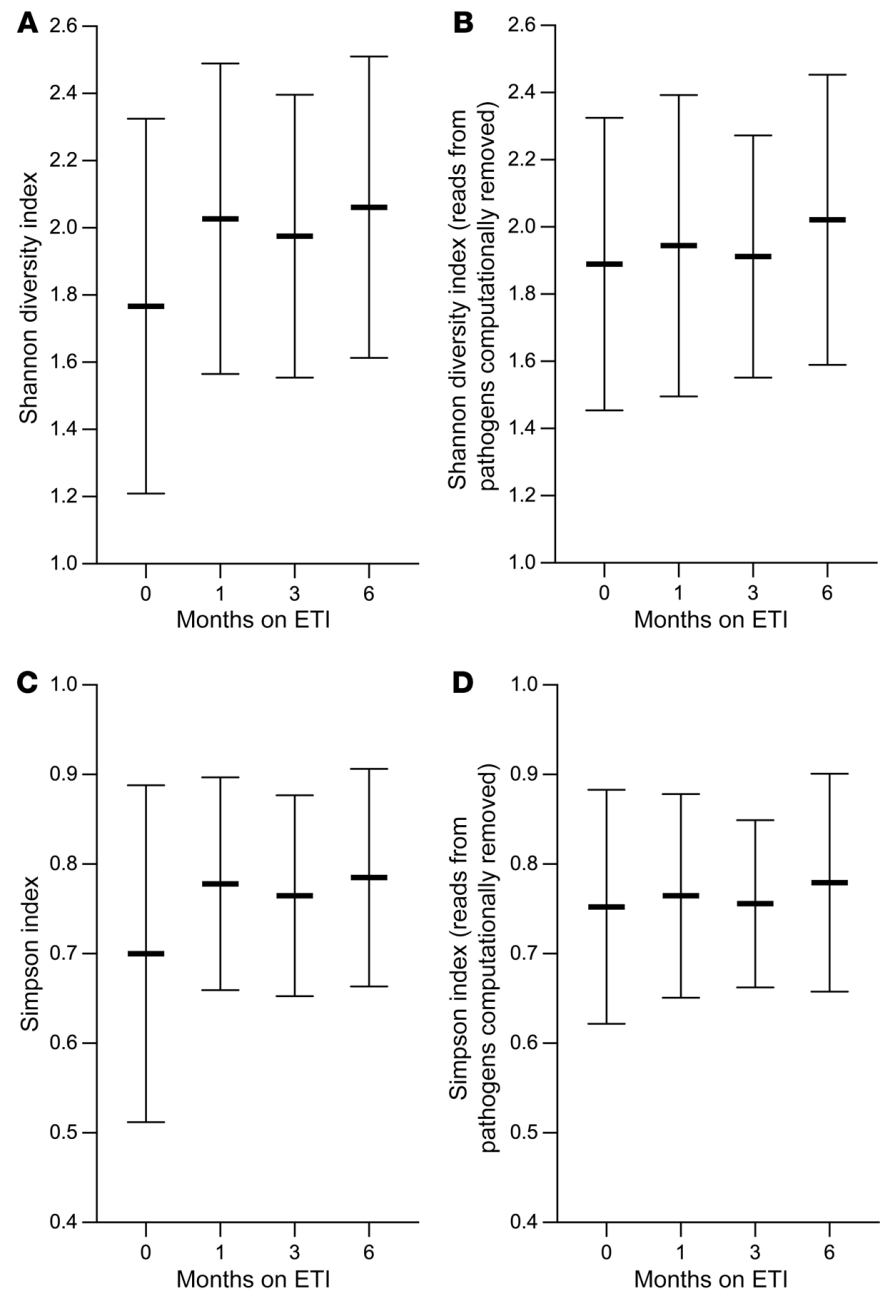
Sputum microbial diversity increases after ETI due to decreases in CF pathogen abundance. (**A** and **C**) Sputum genera relative abundance data were used to calculate the Shannon (**A**) and Simpson (**C**) indices at each time point. (**B** and **D**) To examine the effect CF pathogen abundance changes on the observed increase in diversity, sequencing reads from traditional CF pathogen genera were computationally removed (see text) and the Shannon (**B**) and Simpson (**D**) indices were recalculated. Mean and SD for each time point are shown. There was a significant increase (*P* < 0.05 by 1-way ANOVA) in diversity after ETI compared with before ETI) only when all genera were included; this was true for all post-ETI visits. One hundred forty-five participants were studied at the pre-ETI visit, 123 at 1 month, 56 at 3 months, and 89 at 6 months.

Previous work suggests that the extent of genus-level bacterial diversity within individual sputum samples (i.e., sample α diversity) is a key parameter of lung disease, so we examined ETI’s effects on the Shannon and Simpson diversity indices. Both indices increased 1 month after ETI, and increases were sustained through 6 months (Figure 6, A and C). We investigated the relative contributions of CF pathogens and other bacteria to the increased diversity by computationally removing CF pathogen genera from taxonomic profiles of each sample and found that Shannon and Simpson diversity indices did not change after ETI when these genera were removed (Figure 6, B and D). Thus, the increases in sputum bacterial diversity measured after ETI were caused by decreases in the abundance of CF pathogens rather than changes in density of other bacterial genera.

We also investigated whether ETI produced consistent changes in sputum bacterial composition (i.e., β diversity). We used the Bray-Curtis index (28) that measures pair-wise dissimilarity in the abundances of taxa in each sample relative to all other samples. Identical samples will have a dissimilarity of zero, and samples with completely distinct compositions will have a dissimilarity of 1. We visualized the distribution of samples from each study visit using nonmetric multidimensional scaling (NMDS), which plots them in 2-dimensional space so that samples with similar taxonomic compositions will cluster together, and dissimilar samples will be far apart.

**Figure 7:**
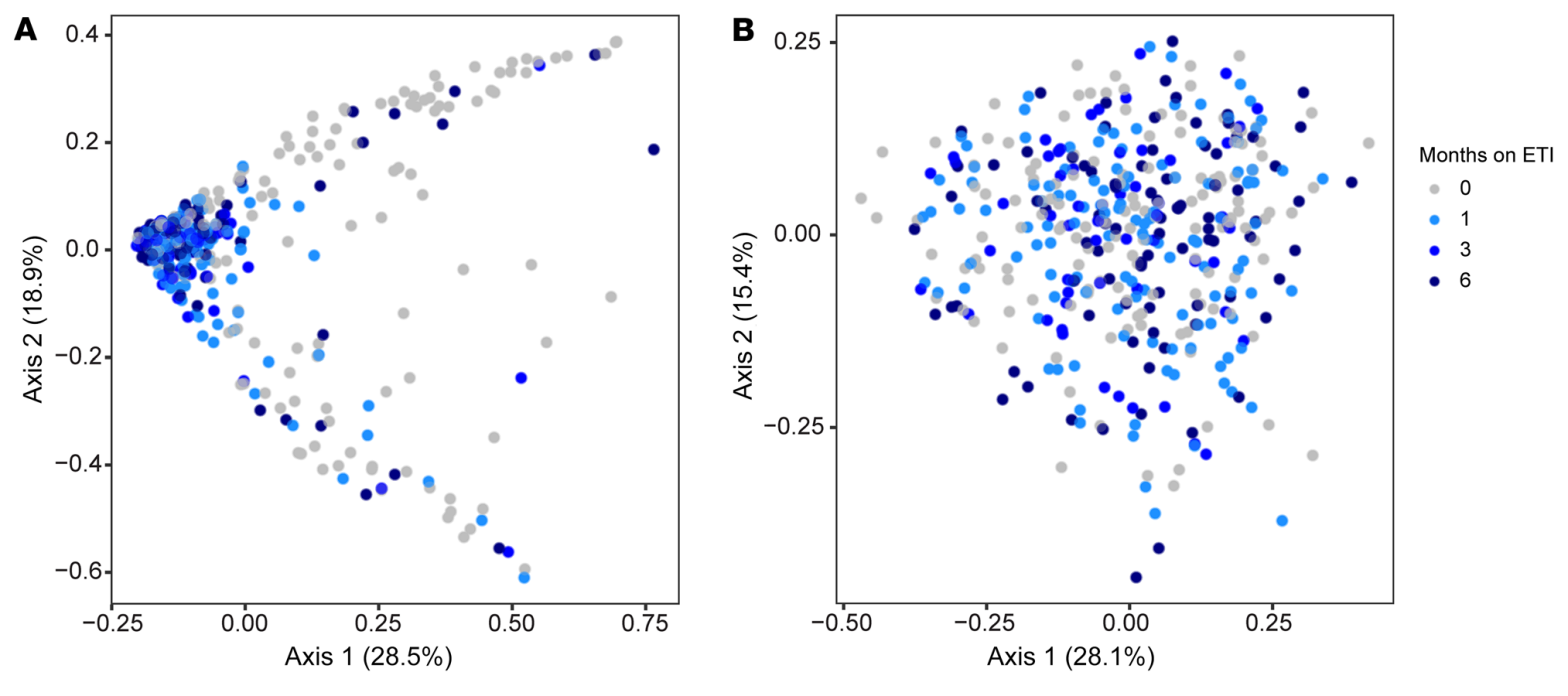
Sputum microbial composition changes after ETI are driven by decreases in traditional CF pathogens. Multidimensional scaling (MDS) representation of the Bray-Curtis index measurements of pair-wise dissimilarity in the abundances of genera in each sputum sample relative to all other samples. Gray indicates samples collected before ETI, light blue 1 month after ETI, blue 3 months after ETI, and dark blue 6 months after ETI. (**A**) Bray-Curtis analysis was performed on all taxonomic read counts from each participant. Pairwise differences between pre-ETI (0 months) and the 1-, 3-, and 6-month visits all *P* < 0.001 by PERMANOVA. (**B**) To examine the effect CF pathogen abundance changes on the shifts in sputum microbial composition, sequencing read counts from traditional CF pathogen genera were computationally removed (see text) and Bray-Curtis pair-wise dissimilarities between samples were recalculated. Pairwise differences between pre-ETI (0 months) and the 1-, 3-, and 6-month visits all not significant by PERMANOVA. One hundred forty-five participants were studied at the pre-ETI time point, 123 at 1 month, 56 at 3 months, and 89 at 6 months.

The NMDS plot showed that pre-ETI samples were found in 2 distinct clusters (gray symbols at the top and bottom of Figure 7A). Biplot analysis of genera contributions to sample positioning in the plots showed that *Pseudomonas* and *Staphylococcus* abundances contributed most to the positioning in the top and bottom pre-ETI visit clusters, respectively (Supplemental Figure 13). ETI initiation led to a marked change in sample distribution, with most post-ETI visits clustering together near the plot’s origin, distinct from pre-ETI visits (Figure 7A) (pairwise differences between pre-ETI and 1-, 3-, and 6-month visits all *P* < 0.001 by PERMANOVA).

However, as observed with α diversity, computationally removing reads corresponding to CF pathogens genera eliminated the pre- and post-ETI differences in population composition (pairwise difference between pre-ETI and 1-, 3-, and 6-month visit not significant by PERMANOVA) (Figure 7B). Similar results were obtained by applying the Horn (29) and Jaccard (30) β diversity indices to the data (Supplemental Figure 14). Together, these analyses show that the pre- to post-ETI change in sputum bacterial population composition is caused by decreases in the abundance of traditional CF pathogens.

## Discussion

Understanding how improved CFTR function affects CF lung infections is critical to defining the nature of the disease for the majority of people with CF now treated with modulators. This interim analysis through 6 months of ETI use produced 4 main findings. First, average sputum densities of traditional CF pathogens, including *S*. *aureus*, *P*. *aeruginosa*, *S*. *maltophilia*, *Achromobacter* spp., and *Burkholderia* spp. all declined by approximately 100-fold or more after 1 month of treatment. Second, a minority of participants who were culture positive at baseline for the most prevalent pathogens (*S*. *aureus* and *P*. *aeruginosa*) became repeatedly culture negative for these pathogens during the first 6 months of treatment. Moreover, DNA from the pathogens present before treatment was found in the sputum of most participants who became culture negative after treatment, raising the possibility that infection persisted despite negative cultures.

Third, DNA-based assays found that other bacteria (organisms not traditionally considered CF pathogens, including *Streptococcus*, *Prevotella*, *Veillonella* spp., and others) were unchanged in abundance in sputum after ETI. Finally, ETI was associated with increased sputum bacterial diversity and produced consistent changes in sputum bacterial composition. However, these changes were caused by decreases in the abundance of traditional CF pathogens, as diversity and composition changes were eliminated when CF pathogen data were computationally removed from the analyses.

### Why does bacterial pathogen density decline after ETI?

Culture- and DNA-based analyses showed that the sputum densities of traditional CF pathogens we measured generally declined to a similar extent and with a similar time course after ETI. This was notable, given the pathogens’ different susceptibilities to innate antimicrobials (31–33) and differences in their mechanisms of pathogenesis and immune evasion (34).

The finding that CF pathogens responded similarly suggests that ETI improves nonspecific host defenses against bacterial infection. While we did not measure host defense function in this study, related research corroborates this interpretation. For example, modulators improve lung mucociliary clearance that indiscriminately clears material deposited in the mucus layer (e.g., bacteria) (7). Modulators also raise airway pH to values closer to those in people without CF (9, 35). This effect is likely to increase the potency of the many antimicrobial factors in airway secretions that have broad-spectrum activity in combination (36). Leukocyte functioning may also be improved after modulators (11, 37, 38). ETI-induced improvements in these relatively nonspecific host defense mechanisms may explain the similar responses observed in organisms with disparate resistance and pathogenesis phenotypes.

### Why did most participants remain infected with CF pathogens?

The finding that a minority of participants with the common pathogens *S*. *aureus* and *P*. *aeruginosa* became repeatedly culture negative during the first 6 months of ETI use is consistent with prior studies of an earlier-generation modulator (ivacaftor) (12, 16). Persistent infection after starting modulators could be explained by several mechanisms. One possible mechanism involves structural lung disease (e.g., bronchiectasis) that may restrict modulator-induced improvement in host defenses to less-diseased lung regions. In support of this idea, the lungs of people with non-CF bronchiectasis exhibit many of the pathological characteristics seen in CF, including damaged surface epithelia, remodeling of submucosal glands and pulmonary vasculature, and airway dilatation (39). This pathology predisposes to chronic infection, with many of the same pathogens seen in CF (40) even though CFTR mutations are absent.

Persistent infection could also be a consequence of abnormalities other than structural damage. For example, longstanding infection can induce an immune-tolerant state that dampens antibacterial responses to pathogens (41) and tolerance could persist after CFTR function is improved. In addition, while ETI is the most active modulator to reach clinical use, it does not fully normalize CFTR function.

Our observation that a small number of participants who were previously culture negative for CF pathogens (based on baseline sputum samples and their prior 2 years of registry data) became culture positive during their first approximately 6 months of ETI treatment is consistent with the idea that susceptibility to some pathogens persists in those initiating ETI in adolescence or adulthood. Longer-term follow-up planned in this study may help us determine whether these infections are transient or become chronic.

Finally, it is possible infection persists after ETI because established infections become autonomous of primary (i.e., CFTR-mediated) or secondary (i.e., lung damage–mediated) host defense defects of CF. Bacterial phenotypes such as slow and aggregated growth (42), high bacterial density, and the genetic adaptation and diversification that occurs during infection (43) can all increase the stress tolerance of pathogens. These and other factors could enable CF infections to persist even if host defenses were completely normalized. The idea that bacterially mediated mechanisms contribute to persistent infection is consistent with our finding that participants who remained *P*. *aeruginosa* culture positive after ETI had markedly higher baseline bacterial densities than those becoming culture negative. High pathogen density may slow growth, cause nutrient limitation, activate bacterial stress responses, and increase the capacity of pathogen populations to adapt to local conditions and genetically diversify (44).

### Why do the sputum densities of CF pathogens decline, while those of other bacteria do not?

Our finding that the sputum densities of traditional CF pathogens decreased by 100- to 1000-fold after ETI, while other bacteria were nearly unchanged, was remarkable. Two general mechanisms could explain this finding. One possibility is that the host defenses improved by ETI are active against traditional CF pathogens but have minimal effects on other bacteria. However, ETI-mediated improvements in mucociliary clearance, mucosal antimicrobial activity, and immune cell function are likely to reduce lung bacteria in a relatively nonspecific fashion. Furthermore, modulator treatment lessens airflow obstruction and reduces mucus plugging (2, 45, 46). These changes should reduce the anaerobic niche postulated to enable growth of *Streptococcus*, *Prevotella*, *Veillonella*, and other anaerobic species in the lung (47). Thus, we think it likely that ETI would nonspecifically reduce bacteria from the lung.

An alternative explanation relates to the fact that sputum contains secretions from the lungs and oropharynx (i.e., saliva). This is important because the major genera we found in sputum that are not traditionally considered CF pathogens (i.e., *Streptococcus*, *Prevotella*, *Veillonella*, *Rothia*, and others) are highly abundant in saliva (48–51). Thus, even modest salivary contamination could have a marked effect on the microbial composition of sputum.

The sputum samples in PROMISE averaged 1 to 2 mL in volume, and DNA-based analyses found that samples contained approximately 10^8^ organisms/mL that are not traditionally considered CF pathogens, both before and after ETI. Nearly all of these organisms are known to be at high abundance in the oral microbiome (48–51). Contamination of sputum with as little as approximately 10% saliva could explain this finding if the salivary abundance of these organisms were approximately 10^9^/mL or higher and were largely unchanged after ETI. Salivary contamination could also explain why total bacterial abundance as measured by 16S rRNA ddPCR did not decline after ETI, as the contribution from high-density bacterial communities in saliva could exceed changes occurring in less abundant CF pathogens originating from the lungs.

Salivary contamination might also explain why induced sputum from healthy individuals contains high numbers of oral bacteria (52–54), while bronchoscopy studies find DNA from these bacteria at very low abundances in healthy lungs (55–57). While uncertain, low-abundance oral bacteria within the lung may occur with recurrent aspiration and immune-mediated killing rather than bacterial replication (55–57). We did not collect or measure bacteria in saliva samples, but many studies find salivary bacterial density in the range of 10^9^ to 10^10^ organisms/mL in both healthy states and disease (48–51, 58). We recognize that some investigators report lower or variable bacterial concentrations in saliva (59), and collection, laboratory, and analytical methods vary between studies.

Two additional points about the potential effects of contamination are noteworthy. First, because the impact of contamination depends on the relative densities of bacteria present in lung versus those originating in the oropharynx (e.g., saliva), ETI’s effect in reducing lung bacteria could amplify the confounding effects of oropharyngeal contamination. Second, contamination could mask changes in organisms that inhabit both the lungs and oropharynx, particularly if the levels of such organisms were much lower in the lung compared with saliva. Our findings on *Haemophilus* exemplifies this problem. While we found little change in *Haemophilus* genus after ETI, further analysis showed that the *Haemophilus* genus signal in sputum was mainly attributable to *Haemophilus* species other than *H*. *influenzae* that are known to be abundant in the oropharynx. Understanding ETI’s effects on organisms that might inhabit both the lungs and oropharynx will likely require direct lung sampling or methods that account for the effects of oropharyngeal contamination.

### Study limitations.

This work has several limitations. First, the study was observational and did not have a control group who were not prescribed ETI. The coincident timing of drug use and sputum pathogen density changes, and consistency of finding across participants suggest that the observed changes occurred because of ETI. In addition, control groups in other CF studies have not exhibited marked declines in sputum pathogen density as observed here (60). Thus, research participation was unlikely to explain the results.

Second, we studied expectorated sputum and sputum analysis has inherent limitations. Sputum may not comprehensively represent lung bacteria, so using sputum to make determinations of the presence and absence of bacteria in lungs is error prone. We partially mitigated this limitation using serial sputum sampling to improve detection, and we did not classify participants as provisionally “repeatedly culture negative” unless all and a minimum of 2 postbaseline samples were negative. Longer follow-up will provide more information. It is also possible that the pathogens detected by ddPCR in culture-negative sputum could have originated from a nonlung compartment (e.g., the oropharynx), or be a different strain than was present before ETI and represent new strain acquisition rather than infection persistence. In addition, since invasive lung sampling and imaging studies were not performed, we could not evaluate the microbiology of those who were unable to produce sputum samples or consider the effects of structural lung disease.

Third, the DNA-based measurements do not distinguish live from dead bacteria, and measurements using 16S rRNA genes (used in relative and calculated absolute abundance measurements) can be inaccurate because organisms vary in 16S copy number. However, the abundant genera focused on here have 16S copy numbers within a similar range, and species-specific PCR that used single-copy targets corroborated key findings.

Fourth, COVID-19 pandemic–related challenges reduced study visit attendance, increased missing data, and necessitated a change from in-clinic sputum induction to spontaneous at-home sputum collection in some participants. Fortunately, the baseline and 1-month visits occurred largely before the pandemic and most of the ETI-associated effects in this and other studies (16) occurred in the first month of treatment.

Finally, the short duration of data reported here (~6 months) makes conclusions about the durability of pathogen reductions, conversion to culture negativity, and onset of “new” infections after ETI provisional. Longer-term follow up in progress will provide important additional data and will also enable us to identify individual characteristics and treatment responses associated with microbiological changes.

### Other work on post-ETI microbiology.

Two published studies have prospectively examined microbiologic changes after ETI. Sosinski and colleagues (61) analyzed sputum from 24 individuals before and at various time points after ETI (range 14–327 days) using 16S rRNA gene sequencing and found that sputum bacterial diversity and evenness increased after ETI. While no individual taxa differed in relative abundance after ETI, the log-ratio CF pathogen relative abundances (summed together) to anaerobes (summed together) significantly decreased. Pallenberg and colleagues (62) compared throat swabs after deep cough from 12 individuals collected before and after ETI and found a decrease in throat bacterial load and an increase in throat bacterial community evenness, which were driven by reductions in *Capnocytophaga* and *Rothia* spp. Differences in the number of people studied, types of samples utilized, and analytical methods employed make it difficult to compare these findings with the work reported here.

### Implications for the future of CF lung disease.

The findings reported here show the beneficial effects of ETI on CF infections and also highlight uncertainties and challenges ahead. On one hand, the pathogen reductions observed after ETI could reduce lung disease progression, and the magnitude of reduction observed could herald marked improvements in prognosis. Recent work suggesting that ETI may improve dysregulated inflammation in CF cells is also encouraging (38), as concurrent reductions in pathogen burden and lung inflammation could have synergistic effects. On the other hand, it is important to recognize that most pathogen-infected participants continued to harbor detectable pathogen burden by culture or PCR after ETI, and that some participants became newly culture positive for traditional CF pathogens while on ETI treatment. Thus, lung infections will likely remain a major focus of CF clinical care and research.

Future work on modulator effects in younger individuals without long-standing infection and less structural lung damage than the participants in this study will provide key prognostic information for early-disease cohorts and shed light on importance of secondary host or bacterial mechanisms in infection persistence. In addition, studies are needed to determine whether additional progress against infection requires further improvements in lung host defenses, new antimicrobial approaches, or both. It will also be critical to determine whether current antibiotic use practices in CF remain beneficial given the marked changes in pathogen burden. Finally, outcomes research is needed to determine the extent to which ongoing infection affects the heath of people with CF treated with modulators.

## Methods

### Study design and participants

The PROMISE core study is a prospective, observational study in 487 people with CF age 12 years or older with at least 1 F508del allele starting ETI for the first time (2). A subset (*n* = 236) of the participants consented to participate in sputum collection for the microbiological analyses reported here (PROMISE-Micro). These participants followed the same study design and eligibility criteria described as the overall cohort. Assessments occurred before and 1, 3, and 6 months during ETI therapy. The primary outcomes were quantitative changes in sputum density of *P*. *aeruginosa* and *S*. *aureus* from baseline assessed by both culture and PCR. Additional outcomes included changes in *S*. *maltophilia*, *Burkholderia* *cepacia* complex, and *Achromobacter* *xylosoxidans* along with changes in bacterial abundance measured by PCR and sequencing. Exploratory studies assessed the rate of conversion to nondetection of key pathogens among those positive at baseline. All PROMISE participants consented to have their study data linked to their CF Foundation Patient Registry (CFFPR) data for historical respiratory microbiology, diagnostic variables, chronic medications, etc., and for future outcomes analysis (63). The study was registered at ClinicalTrials.gov (NCT04038047), and institutional review board approval was granted at each site. See enrollment diagram in Supplemental Figure 15.

#### Sample collection.

Sputum induction was performed by nebulization and inhalation of 7% saline solution. If induction was contraindicated due either to patient health or COVID-19 pandemic policies, then expectorated sputum samples were accepted. Home collection was used for 39 subjects at visit 4 due to COVID-19 pandemic policies; all other samples were collected at a clinic. Samples were shipped overnight on ice.

#### Culture.

Sputum samples were processed at the Center for CF Microbiology (Seattle, Washington, USA) using established protocols (64). Briefly, samples were weighed, and an aliquot was solubilized 1:1 with sputolysin, serially diluted, and plated on MacConkey and mannitol salts agar plates for quantitative culture of *S*. *aureus*, *P*. *aeruginosa*, *S*. *maltophilia*, *Achromobacter* spp., and *Burkholderia* spp. If sample weight was less than 0.1 g, solubilized samples were plated for qualitative culture to determine the presence or absence of bacterial species noted above. Limit of reliable detection for quantitative culture is 60 CFU/g. Unique colony morphologies were identified to species level by mass spectrometry. If fewer than 60 CFU/g (3 colonies) were present but a pathogen detected, the estimated CFU/g was reported. To calculate log reduction in CFU, culture-negative samples were set to half the detection threshold (10 CFU/g).

#### DNA isolation.

A 100 to 200 μL aliquot of solubilized sputum (when volumes were adequate) was stored at –80°C for DNA preparation using the DNeasy Power Soil Pro kit (Qiagen) and the Qiacube automated DNA isolation system (Qiagen). Sputum samples from each participant were extracted using the same reagent batches and at the same time to reduce technical variability, and water was extracted in parallel with sputum samples to serve as a negative control (referred to below as “extraction blanks”). When available, untreated sputum (i.e., sputum not solubilized with DTT prior to freezing) was treated with DNase to degrade DNA from bacteria with permeabilized membranes and human DNA using the QIAmp DNA Microbiome kit (Qiagen) (65). Purified DNA was stored at –80°C prior to PCR and sequencing. Verification of ability of the QIAmp DNA Microbiome kit to remove DNA from dead bacterial cells was performed by treating exponentially growing cells with NaOCl (KIK), polymixen B (Bedford Labs), or Colistin (RPI) for 4 hours prior to DNA extraction of samples with DNeasy Power Soil Pro and QIAmp DNA Microbiome kits. Quantification of bacterial genomes was performed by ddPCR and the difference in genome copies between the extraction methods was compared for each treatment by 1-way ANOVA using PRISM version 9 (GraphPad).

#### ddPCR.

For most samples, ddPCR was performed using 1 μL of 1:100 diluted DNA isolated from solubilized sputum (Bio-Rad QX200 ddPCR system) using the ddPCR Multiplex Supermix (Bio-Rad) and previously validated primer and probe sets (16) adapted for ddPCR. Results were analyzed with QX software to determine target DNA concentrations. For low-abundance samples, up to 13 μL of undiluted DNA was used. Positive control (DNA isolated from bacterial cultures) and negative controls (no sample added) were run with each assay. Results from 16S rRNA gene ddPCR were divided by 4 to correct for 16S rRNA gene copy number. Extraction blanks had a median background level of 1.43 × 10^3^ (range 0 to 3.75 × 10^3^) 16S rRNA gene copies/mL and sputum had a median concentration of 1.3 × 10^8^ gene copies/mL.

ddPCR assays were validated by comparing DNA concentration measurements obtained from serial dilutions of each measured species using Qubit (Thermo Fisher Scientific) and ddPCR (see Supplemental Figure 5). The ddPCR threshold over which pathogens were considered present was set at greater than 200 genomes/mL, as no negative control (i.e., water or extraction blank sample) contained greater than 200 pathogen copies/mL. To calculate log reduction in genome copies/mL, negative samples were set to half the detection threshold (100 copies/mL).

#### 16S rRNA gene sequencing.

Sequencing libraries were prepared in triplicate following the 16S Metagenomic Sequencing Library Preparation protocol (Illumina) targeting V3 and V4 variable regions using 5 μL of DNA from solubilized sputum amplified with Kapa (Roche) using published primers (Supplemental Table 10) that contained Illumina adaptors. Libraries were validated for concentration using Qubit and length using a 2200 TapeStation (Agilent). After indexing and pooling, sequencing was performed on a MiSeq instrument (Illumina) using a 2 × 300 cycle kit. No-template controls (water), extraction blanks, and mock community controls (ZymoBIOMICS Mock Community Standard, Zymo) were used to test the consistency and accuracy of sequencing and data analysis pipeline.

Samples were demultiplexed by MiSeq control software. Reads were filtered and trimmed; denoised, merged, and chimeras removed; and then assigned to amplicon sequence variants (ASVs) using the DADA2 pipeline (66) (version 1.18). ASVs were assigned to the genus level using the SILVA database (67). Any samples with fewer than 20,000 reads after filtering were resequenced. Median read depth after filtering was 100,065 reads for sputum samples and 180 reads for negative controls.

Calculated absolute bacterial abundance was determined by multiplying the total bacterial abundance (16S rRNA genome copies measured by ddPCR) by the relative abundances of each genus ASV (26). Species-specific ddPCR measurements of *P*. *aeruginosa*, *S*. *aureus*, and *S*. *maltophilia* were highly correlated with calculated absolute abundance measurements of the corresponding genus (see Supplemental Figure 11). For analysis purposes, negative values were set to 6.5 × 10^3^ based on the ability to reliably detect low-abundance genera in the mock community controls (ZymoBIOMICS Mock Community Standard II, Zymo) (see Supplemental Figure 10). All sequence read data generated here have been deposited in the Sequence Read Archive at the National Center for Biotechnology Information under BioProject ID: PRJNA934375.

#### MacConkey agar viability assays.

To measure recovery of cultured *P*. *aeruginosa* isolates, 3 *P*. *aeruginosa* isolates from each of 4 participants and the PAO1 reference strain were grown for 40 hours in LB broth (Difco) at 37°C, and then serially diluted and plated onto LB and MacConkey agar. After a 24-hour incubation, CFUs on each media type were determined.

To measure recovery of *P*. *aeruginosa* from sputum, 6 sputum samples from chronically infected participants were shipped overnight on ice, solubilized in DTT, and a 10-fold dilution series plated on LB and MacConkey agar. After 48 hours of growth, recovery of viable bacteria was measured by collecting cells from plates containing more than 200 colonies. ddPCR was used to measure *P*. *aeruginosa* genome copies and total bacterial genome copies (see ddPCR methods above) on DNA made from collected cells. *P*. *aeruginosa* recovery from LB and MacConkey agar was calculated as the *P*. *aeruginosa* genomes/total bacterial genomes × CFU of cultures. Statistical analysis was done with unpaired 2-tailed *t* tests on log-transformed values using PRISM (version 9) software.

#### Analysis of α and β diversity.

Samples with fewer than 1000 16S rRNA sequencing reads were removed and α diversity and Bray-Curtis, Jaccard, and Horn β diversity value, and the Bray-Curtis taxonomic biplot were calculated using the phyloseq package in R using data generated from DADA2 (66, 68). Differences in α diversity between sampling times were calculated by 1-way ANOVA in PRISM (version 9). PERMANOVA tests were calculated using the vegan package in R. MDS plots were generated using the ggplot2 package in R (69). To determine the contribution of pathogens to α and β diversity, pathogen taxa, including Pseudomonas, Staphylococcus, Pandoraea, Stenotrophomonas, Achromobacter, Burkholderia-Caballeronia-Paraburkholderia, Corynebacterium, Corynebacterium_1, Escherichia/Shigella, Haemophilus, and Inquilinus genera were removed from taxonomic profiles using the subset_taxa function in the phyloseq package in R. Measurements of α and β diversity were performed as described above on the remaining taxa in the samples after removing pathogen genera (66, 68).

### Statistics

Summary statistics (i.e., mean, SD, proportion) were used to describe the demographics and baseline characteristics of the overall cohort, and stratified by visits attended. Clinical outcomes and change are presented using summary statistics and paired, 2-tailed *t* tests with 95% CIs at each time point. Chronic medication use, sputum culture collection, pathogen culture prevalence, and *P*. *aeruginosa* cultures after ETI dichotomized by characteristics of participants with *P*. *aeruginosa* detected at baseline were summarized as proportions. “Chronic *P*. *aeruginosa”* was defined using 2 years of prior CFFPR respiratory culture data (70). Participants with at least 3 quarters of *P*. *aeruginosa*–positive cultures in the prior 2 years, and at least 2 quarters with cultures done in each of the 2 years prior to baseline were considered chronically infected. “Not chronic *P*. *aeruginosa*” was defined as less than 3 quarters with positive *P*. *aeruginosa* cultures, and at least 2 quarters with cultures done in each of the 2 years prior to baseline. *P* values for the paired data were generated using McNemar’s exact test. Fisher’s exact test was used to compare *P*. *aeruginosa* cultures after ETI by characteristics of participants with *P*. *aeruginosa* detected at baseline. Repeated measures (i.e., mixed model with random intercept only) analysis was done for the change from baseline in log_10_ pathogen density and log_10_ pathogen absolute abundance. CIs at each time point using mixed-model estimates are from tests based on *t* statistics for estimated change from baseline at the 0.05 significance level. All analyses summarized above were performed with SAS version 9.4 and R version 4.2. Graphs of data were generated using PRISM (version 9). A *P* value of less than 0.05 was considered significant.

### Study approval

The study was registered at ClinicalTrials.gov (NCT04038047), and institutional review board approval was granted at all participating sites.

## Author contributions

DPN, JMV, SLH, JPC, SMR, and PKS conceived, designed, and initiated the study. SJM, MS, SBPS, and SLH performed statistical analysis and provided critical advice. All authors analyzed and interpreted data. DPN and PKS wrote the first draft of the manuscript; all authors provided critical revisions and intellectual content. The PROMISE-Micro Study Group consists of key study team members at the central coordinating center and all lead investigators and research coordinators who are responsible for ethical board approval participant recruitment and conduct at local study sites.

## Supplementary Material

Supplemental data

ICMJE disclosure forms

Supplemental primary data set 1

## Figures and Tables

**Table 2:**
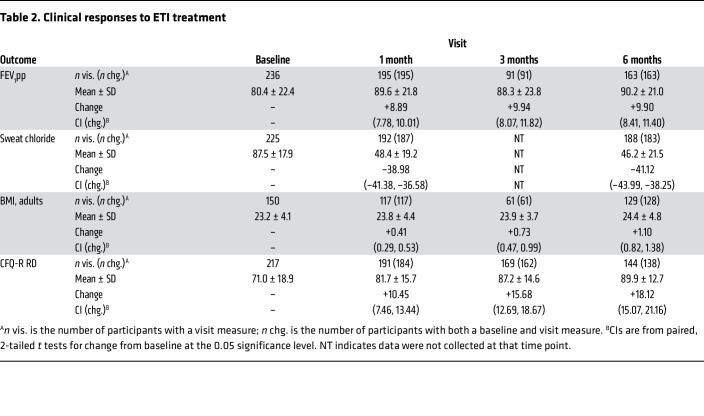
Clinical responses to ETI treatment

**Table 1:**
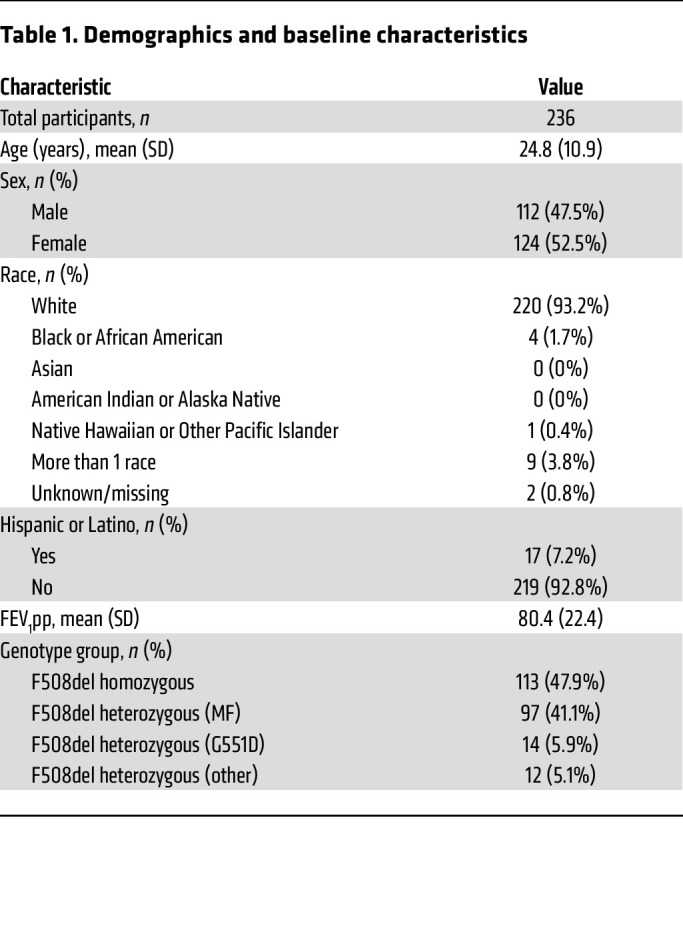
Demographics and baseline characteristics

## Version 1 03/28/2023

In-Press Preview

## Version 2 05/15/2023

Electronic publication
