# Supplementary material for: Pharmacologic improvement of CFTR function rapidly decreases sputum pathogen density, but lung infections generally persist
Source: J Clin Invest. 2023 May 15;133(10):e167957. doi: 10.1172/JCI167957 (PMC10178839; doi:10.1172/JCI167957)

Supplemental Material for:

Pharmacologic Improvement of CFTR Function Rapidly Decreases Sputum Pathogen Density but Lung Infections Generally Persist

David P. Nichols[1], Sarah J Morgan[2], Michelle Skalland[3], Anh T. Vo[2], Jill M. VanDalfsen[3], Sachinkumar B.P. Singh[4], Wendy Ni[2], Lucas Hoffman[1], Kailee McGeer[2], Sonya L. Heltshe[1,3], John P. Clancy[5], Steven M. Rowe[5], Peter Jorth[6], *Pradeep K. Singh[2], and the PROMISE-Micro Study Group

[1]Department of Pediatrics, University of Washington, Seattle, WA USA

[2]Departments of Microbiology and Medicine, University of Washington, Seattle, WA USA

[3]Therapeutics Development Network Coordinating Center, Seattle Children's Research Institute, Seattle, WA USA

[4]Department of Medicine, University of Iowa, Iowa City, IA USA

[5]Cystic Fibrosis Foundation, Bethesda, MD USA

[6]Pathology and Laboratory Medicine, Cedars-Sinai Health System, Los Angeles, CA USA

Supplemental Acknowledgments - The PROMISE-Micro Study Group

Lead Site Investigators:

Omlor, Gregory; Roberts, Dion; Caverly, Lindsay; Hunt, William; Frederick, Carla; Amin, Raouf; McBennett, Kimberly; Taylor-Cousar, Jennifer; Sagel, Scott; Mukadam, Zubin; Schultz, Karen; Lascano, Jorge; Millard, Susan; Graff, Gavin; Mann, Michelle; Braun, Andrew; Antos, Nicholas; Egan, Marie; Walker, Patricia; Mehdi, Nighat; Milla, Carlos; Chittivelu, Subramanyam; Schechter, Michael S.; Aitken, Moira; Gibson, Ronald; Pittman, Jessica; Dozor, Allen J.

Lead Site Research Coordinators:

Parrish, Michelle; Roberts, Vicki; Carey, Jessica; Dangerfield, Joy; Caci, Nadine; Rehn, Kira; Rone, Tia; Wilson, Alix; Cross, Mary; Cahill, James; Finto, Jill; Smith, Alexa; Symington, Thomas; Kitch, Diane; Donado, Mayel; Devereaux, Megan; Roth, Laura; Guzman, Catalina; Demarco, Teresa; Farley, Kim; Spano, Jacquelyn; Scott, Ashley; Lessard, Margaret (Meg); Okhapkina, Olga; Genatossio, Alan; Smith, Sarah; Ramirez, Armando

Coordinating Center and Laboratory Staff:

O'Rourke, Colin; Buccat, Anne Marie; Couture, Laurel; Weaver, Katie; Xie, Jing

Table S1. Demographics and Baseline Characteristics by visits attended

	Overall (N = 236)	Only Baseline Sputum Analyzed (N = 43)	Only Baseline and1 Month Sputum Analyzed (N = 35)	Only Baseline, 1 Month, and 3 Month Sputum Analyzed (N = 15)	Only Baseline, 1 Month, and 6 Month Sputum Analyzed (N = 47)	Sputum Analyzed From All Four Visits (N = 46)
Age (years), mean (SD)	24.8 (10.9)	26.1 (10.6)	24.3 (12.6)	25.2 (9.6)	25.1 (10.9)	27.8 (11.1)
Age (years) distribution, n (%)						
12 to < 18	85 (36.0%)	14 (32.6%)	14 (40.0%)	5 (33.3%)	16 (34.0%)	12 (26.1%)
18 to < 30	79 (33.5%)	14 (32.6%)	10 (28.6%)	5 (33.3%)	16 (34.0%)	12 (26.1%)
30 or older	72 (30.5%)	15 (34.9%)	11 (31.4%)	5 (33.3%)	15 (31.9%)	22 (47.8%)
Sex, n (%)						
Male	112 (47.5%)	20 (46.5%)	12 (34.3%)	7 (46.7%)	26 (55.3%)	16 (34.8%)
Female	124 (52.5%)	23 (53.5%)	23 (65.7%)	8 (53.3%)	21 (44.7%)	30 (65.2%)
Race, n (%)						
White	220 (93.2%)	40 (93.0%)	35 (100%)	15 (100%)	44 (93.6%)	41 (89.1%)
Black or African American	4 (1.7%)	1 (2.3%)	0 (0%)	0 (0%)	1 (2.1%)	1 (2.2%)
Asian	0 (0%)	0 (0%)	0 (0%)	0 (0%)	0 (0%)	0 (0%)
American Indian or Alaska Native	0 (0%)	0 (0%)	0 (0%)	0 (0%)	0 (0%)	0 (0%)
Native Hawaiian or Other Pacific Islander	1 (0.4%)	0 (0%)	0 (0%)	0 (0%)	0 (0%)	0 (0%)
More than One Race	9 (3.8%)	2 (4.7%)	0 (0%)	0 (0%)	2 (4.3%)	3 (6.5%)
Unknown/Missing	2 (0.8%)	0 (0%)	0 (0%)	0 (0%)	0 (0%)	1 (2.2%)
Hispanic or Latino, n (%)						
Yes	17 (7.2%)	2 (4.7%)	1 (2.9%)	2 (13.3%)	5 (10.6%)	3 (6.5%)
No	219 (92.8%)	41 (95.3%)	34 (97.1%)	13 (86.7%)	42 (89.4%)	43 (93.5%)
ppFEV, mean (SD)	80.4 (22.4)	84.7 (22.6)	79.5 (18.3)	76.3 (24.6)	81.2 (21.4)	73.6 (25.0)
ppFEV distribution, n (%)						
< 65	63 (26.7%)	10 (23.3%)	8 (22.9%)	5 (33.3%)	10 (21.3%)	20 (43.5%)
65 to 90	83 (35.2%)	14 (32.6%)	15 (42.9%)	6 (40.0%)	21 (44.7%)	11 (23.9%)
> 90	90 (38.1%)	19 (44.2%)	12 (34.3%)	4 (26.7%)	16 (34.0%)	15 (32.6%)
Height (cm) [18+ y.o.], mean (SD)	167.2 (9.4)	167.6 (10.5)	163.5 (10.9)	166.8 (6.9)	167.3 (8.4)	167.0 (8.4)
Height (percentile) [< 18 y.o.], mean (SD)	41.7 (28.3)	40.0 (27.4)	50.8 (31.3)	16.6 (15.1)	43.2 (27.2)	46.4 (29.5)

	Overall (N = 236)	Only Baseline Sputum Analyzed (N = 43)	Only Baseline and1 Month Sputum Analyzed (N = 35)	Only Baseline, 1 Month, and 3 Month Sputum Analyzed (N = 15)	Only Baseline, 1 Month, and 6 Month Sputum Analyzed (N = 47)	Sputum Analyzed From All Four Visits (N = 46)
Weight (kg) [18+ y.o.], mean (SD)	65.0 (13.8)	64.1 (11.8)	63.1 (12.1)	68.0 (18.6)	66.6 (15.6)	63.8 (13.2)
Weight (percentile) [< 18 y.o.], mean (SD)	51.9 (28.7)	52.4 (29.4)	49.1 (26.8)	36.7 (23.1)	59.7 (28.0)	49.2 (29.9)
BMI (kg/m2) [18+ y.o.], mean (SD)	23.2 (4.1)	22.7 (2.7)	23.6 (3.9)	24.2 (5.2)	23.8 (5.5)	22.8 (3.8)
BMI (percentile) [< 18 y.o.], mean (SD)	56.8 (26.5)	62.1 (24.1)	49.9 (23.1)	56.6 (27.4)	63.3 (25.5)	50.6 (26.9)
Sweat Chloride (mmol/L), mean (SD)	87.5 (17.9)	86.3 (16.8)	92.0 (14.5)	95.4 (14.3)	83.9 (21.1)	86.6 (20.1)
Genotype Group, n (%)						
F508del Homozygous	113 (47.9%)	19 (44.2%)	20 (57.1%)	10 (66.7%)	22 (46.8%)	18 (39.1%)
F508del Heterozygous (MF)	97 (41.1%)	18 (41.9%)	13 (37.1%)	4 (26.7%)	18 (38.3%)	20 (43.5%)
F508del Heterozygous (G551D)	14 (5.9%)	4 (9.3%)	1 (2.9%)	0 (0%)	4 (8.5%)	4 (8.7%)
F508del Heterozygous (other)	12 (5.1%)	2 (4.7%)	1 (2.9%)	1 (6.7%)	3 (6.4%)	4 (8.7%)
Prior Modulator Use, n (%)						
None	119 (50.4%)	24 (55.8%)	16 (45.7%)	5 (33.3%)	22 (46.8%)	24 (52.2%)
Orkambi or Symdeko	104 (44.1%)	17 (39.5%)	18 (51.4%)	10 (66.7%)	20 (42.6%)	18 (39.1%)
Kalydeco	13 (5.5%)	2 (4.7%)	1 (2.9%)	0 (0%)	5 (10.6%)	4 (8.7%)
Chronic Pa+ in 2 Years Prior, n (%)						
Yes	81 (34.3%)	12 (27.9%)	11 (31.4%)	6 (40.0%)	16 (34.0%)	21 (45.7%)
No	127 (53.8%)	20 (46.5%)	20 (57.1%)	7 (46.7%)	28 (69.6%)	22 (47.8%)
Unknown (not enough cultures)	28 (11.9%)	11 (25.6%)	4 (11.4%)	2 (13.3%)	3 (6.4%)	3 (6.5%)

Table S2. Use of medications as a part of chronic therapy at each visit.

Outcome	Visit	Using/Observed (%)	P Value
Inhaled antibiotics	baseline	130/236 (55.1%)	
	1 month	81/195 (41.5%)	
	3 month	47/92 (51.1%)	
	6 month	69/214 (32.2%)	<0.005
Azithromycin	baseline	111/236 (47.0%)	
	1 month	91/195 (46.7%)	
	3 month	39/92 (42.4%)	
	6 month	90/214 (42.1%)	0.12
Hypertonic saline	baseline	184/236 (78.0%)	
	1 month	150/195 (76.9%)	
	3 month	73/92 (79.3%)	
	6 month	147/214 (68.7%)	<0.005
Dornase alfa	baseline	208/236 (88.1%)	
	1 month	178/195 (91.3%)	
	3 month	85/92 (92.4%)	
	6 month	180/214 (84.1%)	0.08

* P value calculated by exact McNemar’s test for paired data at visit 1 and visit 4.

Table S3. Results of sputum collection.

Visit	Sputum collected by participants attending visit*	Induced Sputum Out of All Sputum Collected	Expectorated Sputum Out of All Sputum Collected
Baseline	210/236 (89%)	183/210 (87.1%)	27/210 (12.9%)
1 month	156/195 (80%)	148/156 (94.9%)	8/156 (5.1%)
3 months	71/92 (77.2%)	66/71 (93.0%)	5/71 (7.0%)
6 months	115/215 (53.5%)	49/115 (42.6%)	66/115 (57.4%)

* Attended visits indicates that the participant completed at least some of the visit tasks. Participants could attend visit but not attempt to provide a sputum sample due to clinical contraindications, preference, or COVID-19 restrictions.

Table S4. Average change of traditional CF pathogen density by participant. Only participants with a positive culture for indicated bacteria at baseline were included in analysis.

Pathogen	# of samples	Time Since Baseline	Mean Change From Baseline ± SE	95% Confidence Interval
Staphylococcus aureus	139	Pre-ETI	--	--
	94	1 month	-1.93 ± 0.16	(-2.25, -1.61)
	44	3 months	-1.59 ± 0.21	(-2.01, -1.16)
	71	6 months	-2.05 ± 0.18	(-2.41, -1.7)
Pseudomonas aeruginosa	86	Pre-ETI	--	--
	60	1 month	-1.79 ± 0.26	(-2.3, -1.27)
	33	3 months	-2.22 ± 0.31	(-2.84, -1.59)
	43	6 months	-2.07 ± 0.29	(-2.64, -1.5)
Stenotrophomonas maltophilia	38	Pre-ETI	--	--
	26	1 month	-3.04 ± 0.37	(-3.8, -2.28)
	14	3 months	-3.29 ± 0.42	(-4.15, -2.44)
	23	6 months	-2.73 ± 0.38	(-3.5, -1.95)
Achromobacter species	11	Pre-ETI	--	--
	10	1 month	-2.61 ± 0.72	(-4.24, -0.97)
	4	3 months	-2.31 ± 0.85	(-4.24, -0.38)
	7	6 months	-1.78 ± 0.77	(-3.52, -0.04)
Burkholderia species	7	Pre-ETI	--	--
	7	1 month	-1.33 ± 0.51	(-2.75, 0.09)
	2	3 months	-2.45 ± 0.81	(-4.71, -0.18)
	4	6 months	-2.45 ± 0.63	(-4.19, -0.7)

* Repeated measures analysis. Estimates are from a mixed model (random intercept only model). This excludes any participants that did not have a positive quantitative value at baseline or did not have any post-baseline quantitative values available. Confidence intervals are from tests based on t-statistic for estimated change from baseline at the 0.05 significance level. P-value was <0.0001 for *S. aureus, P. aeruginosa, and S. maltophilia*. For analysis purposes species which were not detected were set to 10 (see methods for more information).

Table S5: Comparison of rates of clearance between participants who gave two post-ETI samples and participants who gave all three post-ETI samples

	# Participants that became repeatedly negative (only two post-ETI cultures) (%)	# Participants that became repeatedly negative (three post-ETI cultures) (%)
S. aureus	3/52 (5.8%)	3/31 (10%)
P. aeruginosa	7/25 (28%)	8/24 (33.3%)
S. maltophilia	8/14 (64%)	8/11 (73%)
Achromobacter	1/3 (33.3%)	2/4 (50%)
Burkholderia	1/2 (50%)	0/2 (0%)

Table S6. **Proportion of participants not analyzed for conversion to "repeatedly culture-negative" status because of failed sputum induction.**

**Pre-ETI culture results**	**Unanalyzable due to failed induction(s)* (%)**
All Participants	19/206 (9.2%)
S. aureus	17/154 (11%)
P. aeruginosa	5/90 (5.6%)
S. maltophilia	0/39 (0%)
Achromobacter	0/12 (0%)
Burkholderia	1/8 (12.5%)

* Participants were considered to have "failed induction" if the study site reported that sputum induction was attempted, but no sputum was produced.

Table S7. Sensitivity analysis wherein those with failed sputum inductions are treated as culture-negative sputum for analysis of conversion to "repeatedly culture-negative" status.

Pre-ETI culture result	Repeatedly negative/ total* (%)	Repeatedly negative or failed induction/total* (%)
S. aureus	5/83 (6.0%)	11/100 (11%)
P. aeruginosa	14/50 (28.0%)	15/55 (27.3%)
S. maltophilia	16/25 (64%)	16/25 (64.0%)
Achromobacter	3/7 (42.9%)	3/7 (42.9%)
Burkholderia	1/4 (25.0%)	2/5 (40%)

* Participants were considered "repeatedly culture-negative" if they were culture-positive for the indicated pathogen pre-ETI, they produced sputum at least two post-ETI visits, and all (and at least two) samples collected after ETI were culture-negative for the pathogen present pre-treatment. This definition is used in the main text.

** Sputum samples that were not collected due to attempted but failed sputum induction were counted as culture-negative. Thus, participants were included if they were positive for the indicated pathogen pre-ETI and either all (and at least two) samples collected after ETI were culture-negative for the pathogen present pre-treatment; or if all samples collected after ETI were culture-negative for the pathogen present pre-treatment and the sum of collected samples and sputum induction attempts was two or more.

Table S8. Chronic, mucoidy, and density of Baseline* *P. aeruginosa* (Pa) * versus repeated negative *P. aeruginosa* cultures post ETI.

		Pa Culture Pattern after baseline[a]	
**Characteristic**		Repeatedly Pa culture negative after baseline	Not repeatedly Pa culture negative after baseline
Chronic Pa[b] (n=47 evaluated)	Chronic Pa, n=40	10/40 (25.0%)	30/40 (75.0%)
	Not Chronic Pa, n=7	4/7 (57.1%)	3/7 (42.9%)
	P-Value[c]	0.173	
Mucoidy Pa (n=50 evaluated)	Any Mucoid Pa, n=36	8/36 (22.2%)	28/36 (77.8%)
	No Mucoid Pa, n=14	6/14 (42.9%)	8/14 (57.1%)
	P-Value[c]	0.173	
Pa Density (n=48 evaluated)	Lowest tertile, n=16 (CFU <= 4.2×10^4)	8/16 (50%)	8/16 (50%)
	Middle tertile, n=17 (4.2×10^4 < CFU <= 1.1×10^7)	5/17 (29.4%)	12/17 (70.6%)
	Highest tertile, n=15 (CFU > 1.1×10^7)	0/15 (0%)	15/15 (100%)
	P-Value[c]	0.005	
Starting FEV1 percent predicted[d] (n=50 evaluated)	Lowest tertile, n=17 (FEV < 60)	4/17 (23.5%)	13/17 (76.5%)
	Middle tertile n=16 (62 < FEV <79.6)	3/16 (18.8%)	13/16 (81.3%)
	Highest tertile n=17 (FEV > 79.7)	7/17 (41.2%)	10/17 (58.8%)
	P-Value[c]	0.384	
Age at pre-ETI visit (n=50 evaluated)	Youngest tertile n=17 (< 24 years)	8/17 (47.1%)	9/17 (52.9%)
	Middle tertile n=16 (24 < years < 34.3)	4/16 (25%)	12/16 (75%)
	Oldest tertile n=17 (> 34.6 years)	2/17 (11.8%)	15/17 (88.2%)
	P-Value[c]	0.079	
Maintanance of Inhaled Antibiotics[e] (n=41 evaluated)	Continued Inhaled Antibiotics n=27	7/27 (25.9%)	20/27 (74.1%)
	Stopped Inhaled Antibiotics n=14	4/14 (28.6%)	10/14 (71.4%)
	P-Value[c]	>0.999	
Maintanance of Hypertonic Saline[e] (n=40 evaluated)	Continued Hypertonic Saline n=29	10/29 (34.5%)	19/29 (65.5%)
	Stopped Hypertonic Saline	2/11 (18.2%)	9/11 (81.8%)
	P-Value[c]	0.451	

[a] Category includes participants that were positive for Pa at baseline and had at least two post-baseline culture results available.

[b] Pa chronicity was determined in participants for whom there were at least two quarters with culture results per year in the two years prior to the study. Chronic Pa Status was defined as at least 3 quarters Pa positive in the two years prior to the study.

[c] The P-value is from a Fisher's Exact test.

[d] Change in FEV percent predicted determined by pre-ETI compared to 6-months post-ETI

e Continuation of maintenance antibiotic treatment and hypertonic saline was determined for participants who entered the study on that treatment and treatment 6-months post-ETI. Data is based on participant self-reporting.

Table S9. Change in genera calculated absolute abundance for participants with genera detected at baseline

Genera (# positive pre-ETI)	# of samples	Time Since Baseline	Mean Change From Baseline ± SD	95% Confidence Interval	P Value
Staphylococcus (115)	115	Pre-ETI	--	--	--
	94	1 month	-1.81 ± 0.15	(-2.11, -1.52)	<0.0001
	41	3 months	-1.48 ± 0.19	(-1.86, -1.1)	<0.0001
	68	6 months	-1.73 ± 0.16	(-2.05, -1.41)	<0.0001
Pseudomonas (70)	70	Pre-ETI	--	--	--
	60	1 month	-1.32 ± 0.14	(-1.6, -1.04)	<0.0001
	32	3 months	-1.53 ± 0.17	(-1.86, -1.19)	<0.0001
	44	6 months	-1.72 ± 0.15	(-2.03, -1.42)	<0.0001
Stenotrophomonas (21)	21	Pre-ETI	--	--	--
	20	1 month	-2.3 ± 0.23	(-2.78, -1.82)	<0.0001
	11	3 months	-2.35 ± 0.29	(-2.94, -1.76)	<0.0001
	17	6 months	-2.05 ± 0.24	(-2.55, -1.54)	<0.0001
Haemophilus (99)	99	Pre-ETI	--	--	--
	78	1 month	-0.39 ± 0.12	(-0.62, -0.16)	0.0013
	36	3 months	-0.26 ± 0.17	(-0.59, 0.07)	0.1244
	52	6 months	-0.22 ± 0.14	(-0.5, 0.06)	0.1173
Escherichia/Shigella (7)	7	Pre-ETI	--	--	--
	6	1 month	-1.55 ± 0.41	(-2.69, -0.41)	0.0194
	2	3 months	-0.66 ± 0.71	(-2.63, 1.31)	0.4069
	5	6 months	-1.64 ± 0.45	(-2.88, -0.39)	0.0219
Streptococcus (145)	145	Pre-ETI	--	--	--
	118	1 month	-0.11 ± 0.05	(-0.21, 0)	0.0422
	56	3 months	0.02 ± 0.07	(-0.13, 0.16)	0.8131
	85	6 months	0.06 ± 0.06	(-0.06, 0.18)	0.3157
Prevotella (143)	143	Pre-ETI	--	--	--
	116	1 month	-0.22 ± 0.07	(-0.37, -0.08)	0.0033
	55	3 months	-0.04 ± 0.1	(-0.24, 0.16)	0.6723
	83	6 months	0.25 ± 0.08	(0.08, 0.42)	0.0036
Veillonella (141)	141	Pre-ETI	--	--	--
	116	1 month	-0.13 ± 0.06	(-0.26, -0.01)	0.0415
	54	3 months	0.11 ± 0.09	(-0.07, 0.29)	0.2118
	82	6 months	0.16 ± 0.07	(0.02, 0.31)	0.0303
Gemella (129)	129	Pre-ETI	--	--	--
	106	1 month	-0.13 ± 0.09	(-0.3, 0.04)	0.1337
	48	3 months	-0.05 ± 0.12	(-0.28, 0.19)	0.7023
	76	6 months	0.18 ± 0.1	(-0.02, 0.37)	0.0756
Granulicatella (131)	131	Pre-ETI	--	--	--
	108	1 month	-0.19 ± 0.08	(-0.34, -0.04)	0.0146
	50	3 months	-0.18 ± 0.11	(-0.39, 0.03)	0.0991
	74	6 months	0.03 ± 0.09	(-0.15, 0.21)	0.7519
Actinomyces (129)	129	Pre-ETI	--	--	--

Genera (# positive pre-ETI)	# of samples	Time Since Baseline	Mean Change From Baseline ± SD	95% Confidence Interval	P Value
	106	1 month	-0.08 ± 0.07	(-0.22, 0.05)	0.2308
	51	3 months	-0.12 ± 0.09	(-0.3, 0.07)	0.2026
	73	6 months	0.22 ± 0.08	(0.07, 0.38)	0.0058
Porphyromonas (113)	113	Pre-ETI	--	--	--
	92	1 month	-0.4 ± 0.09	(-0.59, -0.21)	0.0001
	42	3 months	-0.27 ± 0.13	(-0.54, -0.01)	0.0451
	64	6 months	-0.04 ± 0.11	(-0.26, 0.18)	0.6918
Neisseria (96)	96	Pre-ETI	--	--	--
	76	1 month	-0.26 ± 0.11	(-0.49, -0.03)	0.0247
	33	3 months	-0.2 ± 0.17	(-0.54, 0.14)	0.2558
	54	6 months	-0.19 ± 0.13	(-0.45, 0.08)	0.1726
Rothia (67)	67	Pre-ETI	--	--	--
	59	1 month	-0.48 ± 0.1	(-0.69, -0.28)	<0.0001
	28	3 months	-0.42 ± 0.14	(-0.69, -0.14)	0.0034
	46	6 months	-0.28 ± 0.11	(-0.51, -0.05)	0.0174

Genera (# positive pre-ETI)	# of samples	Time Since Baseline	Mean Change From Baseline ± SD	95% Confidence Interval	P Value
Staphylococcus (115)	115	Pre-ETI	--	--	--
	94	1 month	-1.81 ± 0.15	(-2.11, -1.52)	<0.0001
	41	3 months	-1.48 ± 0.19	(-1.86, -1.1)	<0.0001
	68	6 months	-1.73 ± 0.16	(-2.05, -1.41)	<0.0001
Pseudomonas (70)	70	Pre-ETI	--	--	--
	60	1 month	-1.32 ± 0.14	(-1.6, -1.04)	<0.0001
	32	3 months	-1.53 ± 0.17	(-1.86, -1.19)	<0.0001
	44	6 months	-1.72 ± 0.15	(-2.03, -1.42)	<0.0001
Stenotrophomonas (21)	21	Pre-ETI	--	--	--
	20	1 month	-2.3 ± 0.23	(-2.78, -1.82)	<0.0001
	11	3 months	-2.35 ± 0.29	(-2.94, -1.76)	<0.0001
	17	6 months	-2.05 ± 0.24	(-2.55, -1.54)	<0.0001
Haemophilus (99)	99	Pre-ETI	--	--	--
	78	1 month	-0.39 ± 0.12	(-0.62, -0.16)	0.0013
	36	3 months	-0.26 ± 0.17	(-0.59, 0.07)	0.1244
	52	6 months	-0.22 ± 0.14	(-0.5, 0.06)	0.1173
Escherichia/Shigella (7)	7	Pre-ETI	--	--	--
	6	1 month	-1.55 ± 0.41	(-2.69, -0.41)	0.0194
	2	3 months	-0.66 ± 0.71	(-2.63, 1.31)	0.4069
	5	6 months	-1.64 ± 0.45	(-2.88, -0.39)	0.0219
Streptococcus (145)	145	Pre-ETI	--	--	--
	118	1 month	-0.11 ± 0.05	(-0.21, 0)	0.0422
	56	3 months	0.02 ± 0.07	(-0.13, 0.16)	0.8131
	85	6 months	0.06 ± 0.06	(-0.06, 0.18)	0.3157
Prevotella (143)	143	Pre-ETI	--	--	--
	116	1 month	-0.22 ± 0.07	(-0.37, -0.08)	0.0033
	55	3 months	-0.04 ± 0.1	(-0.24, 0.16)	0.6723
	83	6 months	0.25 ± 0.08	(0.08, 0.42)	0.0036

Genera (# positive pre-ETI)	# of samples	Time Since Baseline	Mean Change From Baseline ± SD	95% Confidence Interval	P Value
Veillonella (141)	141	Pre-ETI	--	--	--
	116	1 month	-0.13 ± 0.06	(-0.26, -0.01)	0.0415
	54	3 months	0.11 ± 0.09	(-0.07, 0.29)	0.2118
	82	6 months	0.16 ± 0.07	(0.02, 0.31)	0.0303
Gemella (129)	129	Pre-ETI	--	--	--
	106	1 month	-0.13 ± 0.09	(-0.3, 0.04)	0.1337
	48	3 months	-0.05 ± 0.12	(-0.28, 0.19)	0.7023
	76	6 months	0.18 ± 0.1	(-0.02, 0.37)	0.0756
Granulicatella (131)	131	Pre-ETI	--	--	--
	108	1 month	-0.19 ± 0.08	(-0.34, -0.04)	0.0146
	50	3 months	-0.18 ± 0.11	(-0.39, 0.03)	0.0991
	74	6 months	0.03 ± 0.09	(-0.15, 0.21)	0.7519
Actinomyces (129)	129	Pre-ETI	--	--	--
	106	1 month	-0.08 ± 0.07	(-0.22, 0.05)	0.2308
	51	3 months	-0.12 ± 0.09	(-0.3, 0.07)	0.2026
	73	6 months	0.22 ± 0.08	(0.07, 0.38)	0.0058
Porphyromonas (113)	113	Pre-ETI	--	--	--
	92	1 month	-0.4 ± 0.09	(-0.59, -0.21)	0.0001
	42	3 months	-0.27 ± 0.13	(-0.54, -0.01)	0.0451
	64	6 months	-0.04 ± 0.11	(-0.26, 0.18)	0.6918
Neisseria (96)	96	Pre-ETI	--	--	--
	76	1 month	-0.26 ± 0.11	(-0.49, -0.03)	0.0247
	33	3 months	-0.2 ± 0.17	(-0.54, 0.14)	0.2558
	54	6 months	-0.19 ± 0.13	(-0.45, 0.08)	0.1726
Rothia (67)	67	Pre-ETI	--	--	--
	59	1 month	-0.48 ± 0.1	(-0.69, -0.28)	<0.0001
	28	3 months	-0.42 ± 0.14	(-0.69, -0.14)	0.0034
	46	6 months	-0.28 ± 0.11	(-0.51, -0.05)	0.0174

* Repeated measures analysis. Estimates are from a mixed model (random intercept only model). This excludes any participants that did not have a positive quantitative value at baseline or did not have any post-baseline quantitative values available. Confidence intervals from tests based on t-statistic for estimated change from baseline at the 0.05 significance level. For analysis purposes genera which were not detected were set to 6500 (see methods for more information).

Table S10. **PCR primers used in this study**

Primer set	Sequence
16S PCR for sequencing	Forward: TCGTCGGCAGCGTCAGATGTGTATAAGAGACAGCCTACGGGNGGCWGCAG<br>Reverse: GTCTCGTGGGCTCGGAGATGTGTATAAGAGACAGGACTACHVGGGTATCTAATCC
P. aeruginosa ddPCR	Forward: CCGTGGTGGTAGACCTGTTCCCAGACC<br>Reverse: CGCAGCAGGATGCCGACGCC<br>Probe: CCGTGGTGGTAGACCTGTTCCCAGACC
S. aureus ddPCR	Forward: GCGATTGATGGTGATACGGTT<br>Reverse: AGCCAAGCCTTGACGAACTAAAGC<br>Probe: GGTGTAGAGAAATATGGTCCTGAAGCAAGT
S. maltophilia ddPCR	Forward: AAGGACAAGGCGATGACCATC<br>Reverse: CCCCACCACGAYTTCATCA<br>Probe: CAGAACGACATCTGGTTGGCG
16S ddPCR	Forward: TCCTACGGGAGGCAGCAG<br>Reverse: GGACTACCAGGGTATCTAATCCTGTT<br>Probe: CGTATTACCGCGGCTGCTGGCA

Supplementary Figures and Legends

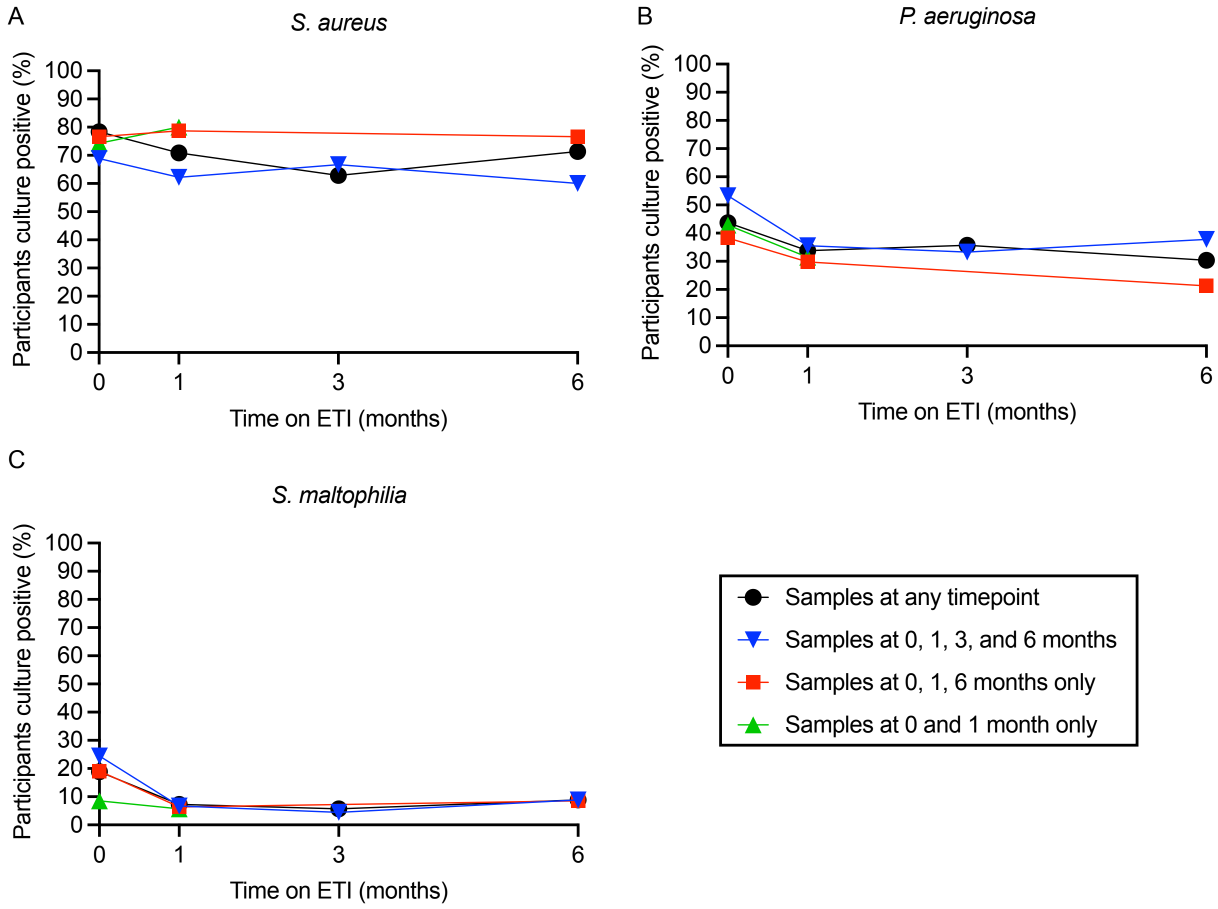

Figure S1. Sputum culture positivity after ETI.

Change in the proportion of participants providing sputum samples that were culture-positive for *P. aeruginosa* (A), *S. aureus* (B), or *S. maltophilia* (C) based on data availability by visit. Blue lines represent participants providing sputum at all study visits; red lines represent participants providing sputum at 0, 1, and 6 months (but not at 3 mo); green lines represent participants providing sputum sample at 0 and 1 month (but not at 3 and 6 mo); black lines represent all participants including those missing data at one or more study visit (this data is also represented in Fig 1b and reproduced here for comparison).

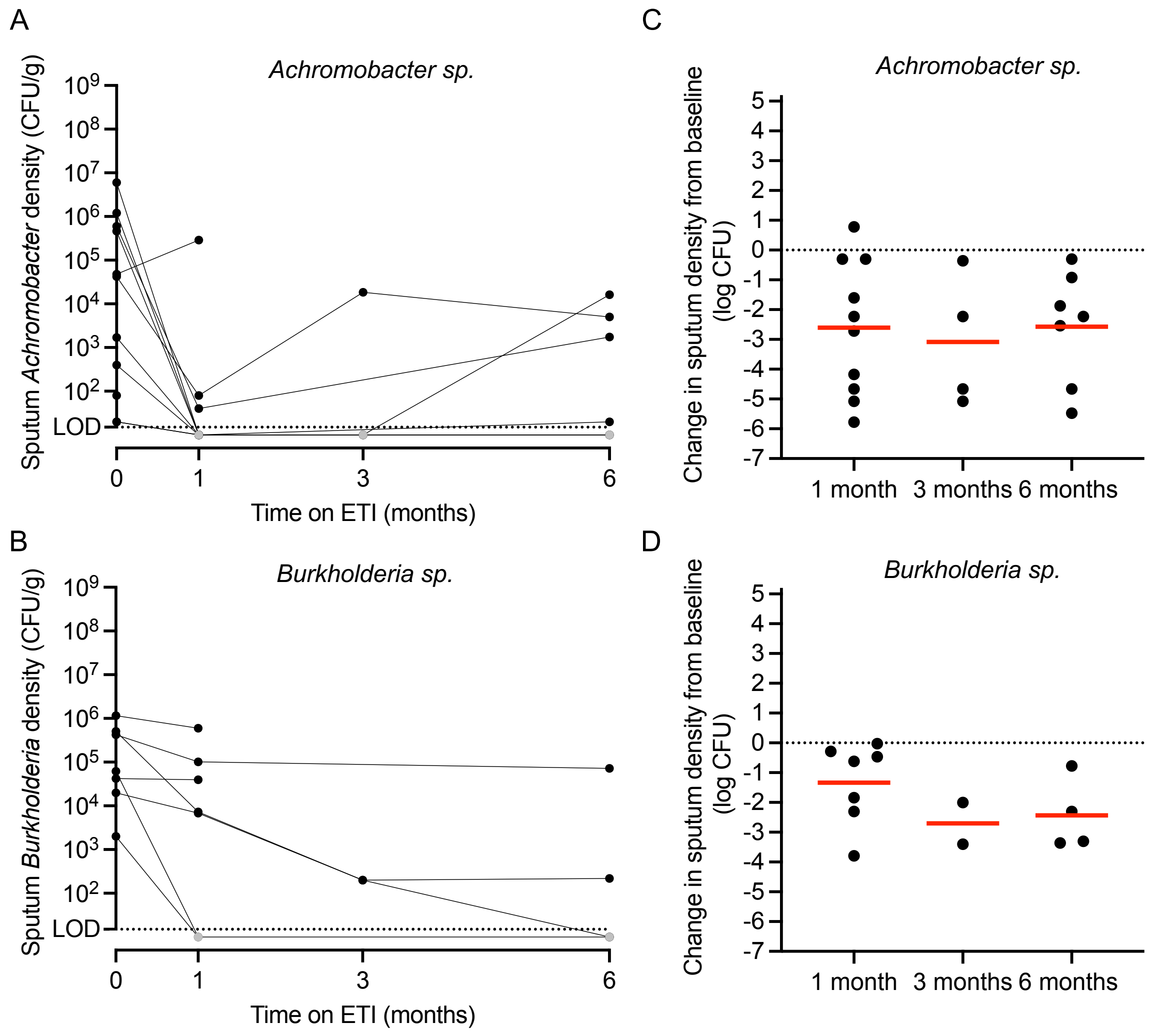

Figure S2. Sputum density of CF pathogens declines after ETI.

A-B. Density of indicated pathogens measured by quantitative culture for participants who were culture-positive for the indicated pathogen at the baseline visit. Data from individual participants are indicated in black, averages are indicated in red. The limit of detection was 20 cfu/ml (dotted line).

C-D. By participant changes in sputum density of indicated pathogens measured by quantitative culture for participants who were culture-positive for the indicated pathogen at the baseline visit. Data from individual participants are indicated in black, averages are indicated in red. See table S4 for average values.

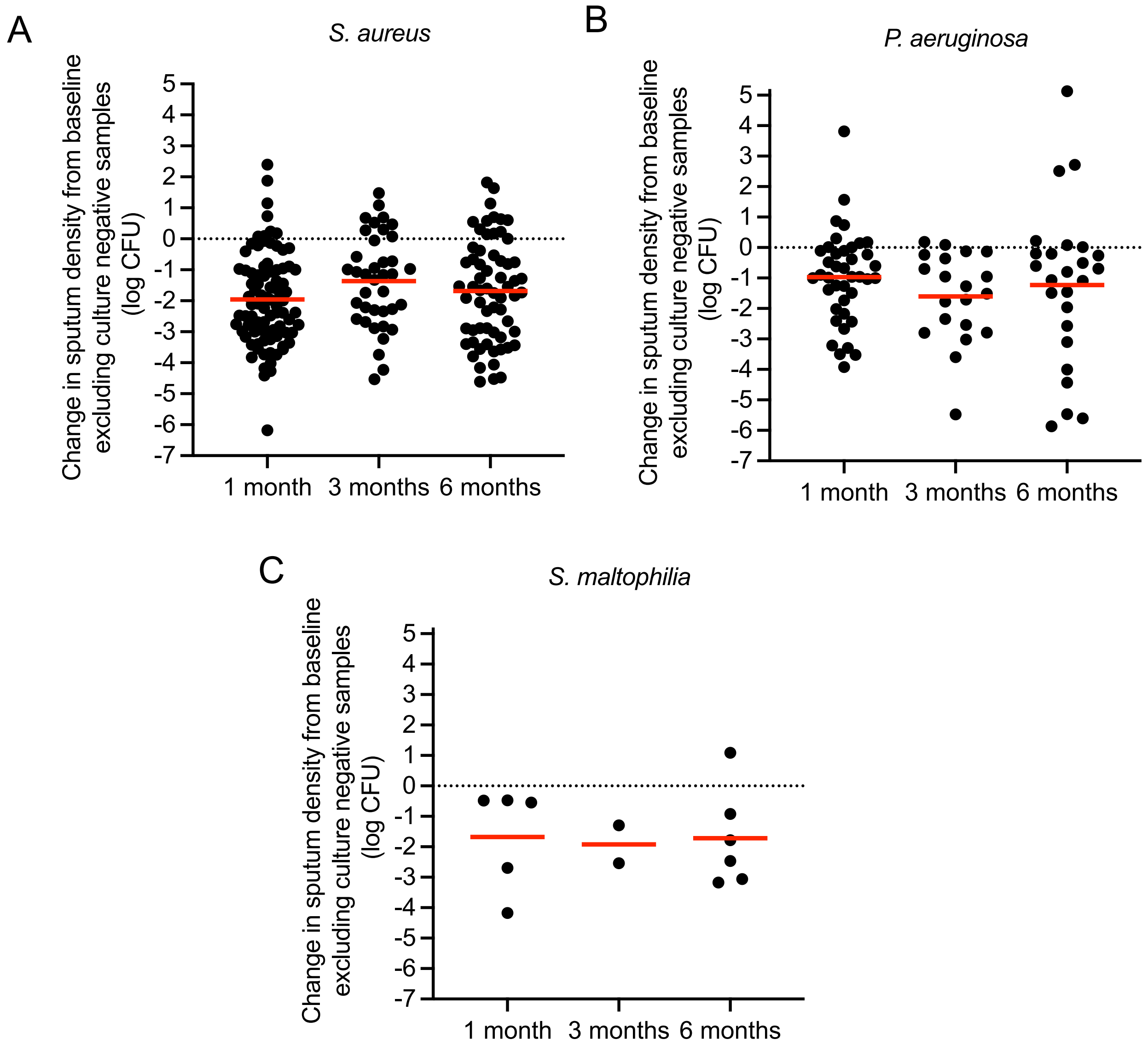

Figure S3. Sputum density of CF pathogens declines after ETI in participants who remained culture positive.

A-C. By participant changes in sputum density of indicated pathogens measured by quantitative culture for participants who were culture-positive for the indicated pathogen at the baseline visit. Data from individual participants are indicated in black, averages are indicated in red.

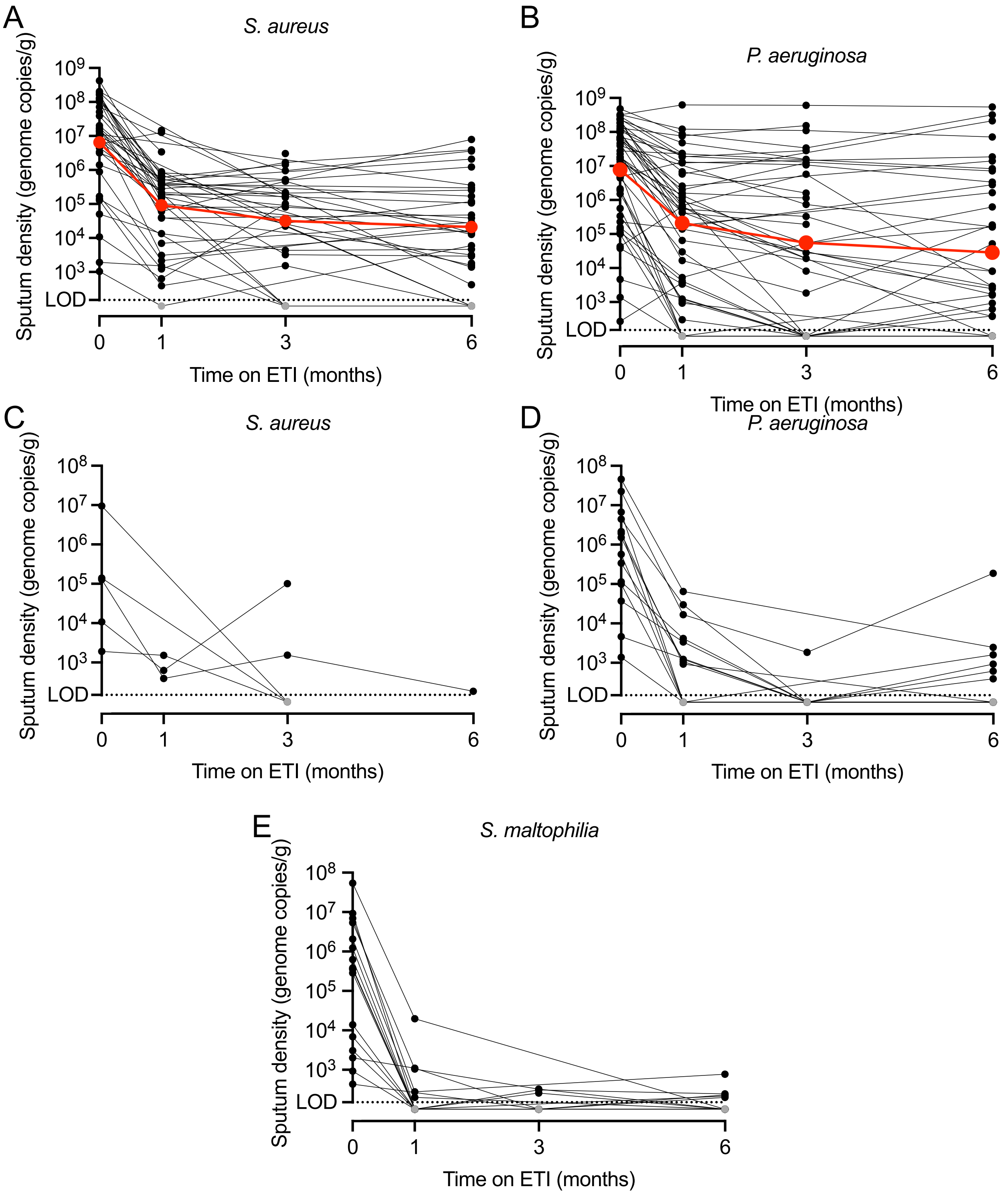

Figure S4. Droplet digital PCR measurements of CF pathogens in sputum.

A&B. Species specific droplet digital PCR (ddPCR) measurements of *S. aureus* (A) and *P. aeruginosa* (B) for participants who were culture-positive for the indicated pathogen at the baseline visit (see Fig 3 for quantitative culture results and Fig S4 for culture positivity results for these participants) Data from individual participants

are indicated in black, geometric mean of data are indicated in red; limit of detection was 200 genome copies/ml (See methods).

C-E. Species specific droplet digital PCR (ddPCR) measurements of *S. aureus* (A), *P. aeruginosa* (B) and *S. maltophilia* (C) abundance in sputum of participants that became repeatedly culture negative for indicted organism (i.e. participants had positive baseline cultures, but all subsequent cultures were negative for indicated organism). Data from these participants are included in panels A&B above.

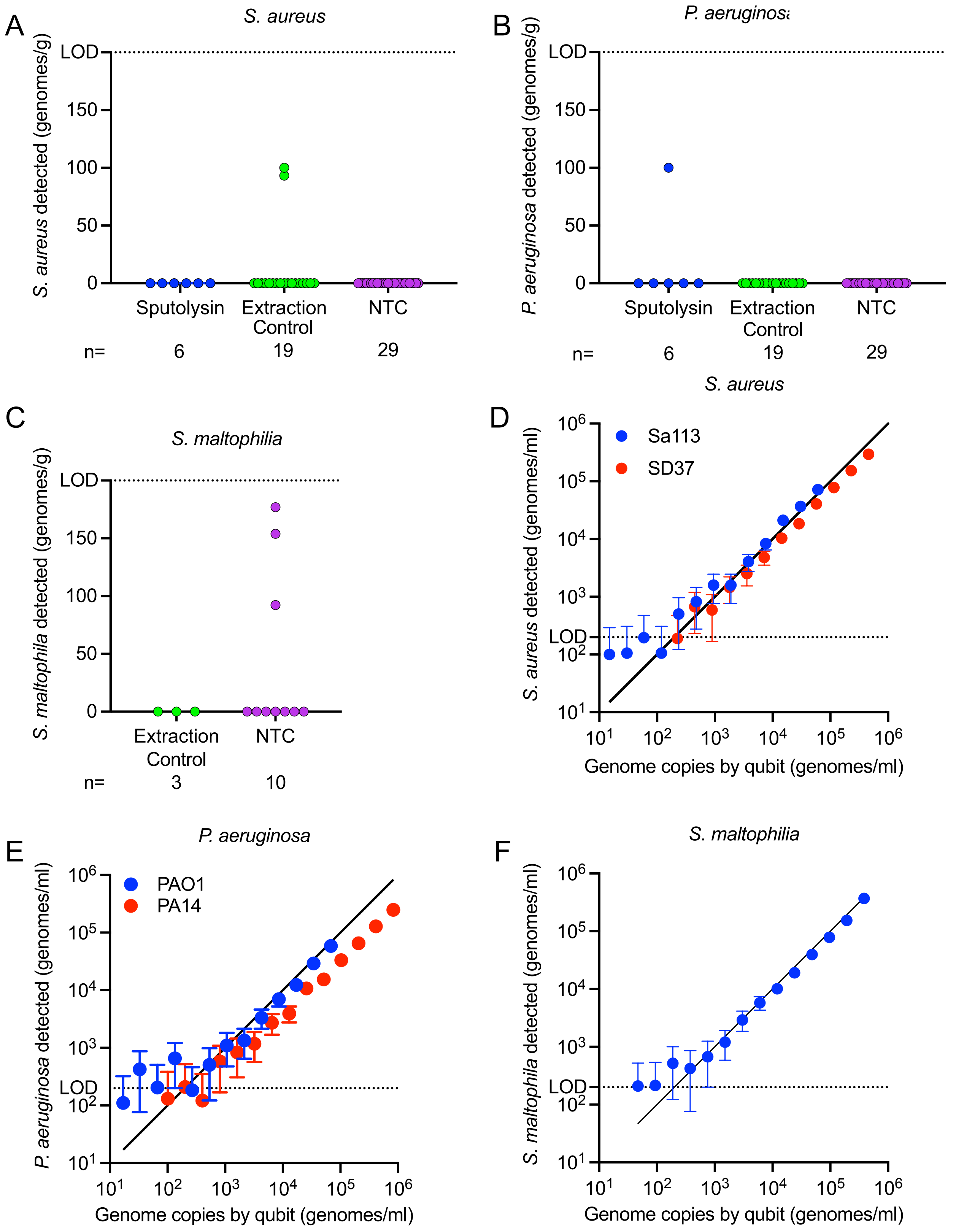

Figure S5. Negative and positive controls for ddPCR assays.

A-C. Negative controls for species-specific ddPCR assays of *S. aureus* (A), *P. aeruginosa* (B), and *S. maltophilia* (C). "Sputolysin" indicates results on aliquots of sputolysin used to dilute sputum' "Extraction

controls" indicate results on water after DNA extraction processing, and "NTC" indicate no template controls of ddPCR performed on water. Detection limits for ddPCR used in this study are indicated by a dashed line.

D-F. Accuracy and detection limits for ddPCR assays. Cultures of *S. aureus* (D), *P. aeruginosa* (E), or S. *maltophilia* (F) were diluted serially 2-fold and bacterial abundance measured by species-specific ddPCR. DNA concentrations present in the undiluted cultures was measured by fluorimetry (Quibit, Invitrogen) enabling estimation of the genome copies/ml in each dilution (indicated on the X-axis). Red and blue points for *S. aureus* (D) and *P. aeruginosa* (E) show species-specific ddPCR results for two different reference strains. The black line indicates a 1:1 correlation between estimated and ddPCR-measured values. Dashed line indicates limits of detection.

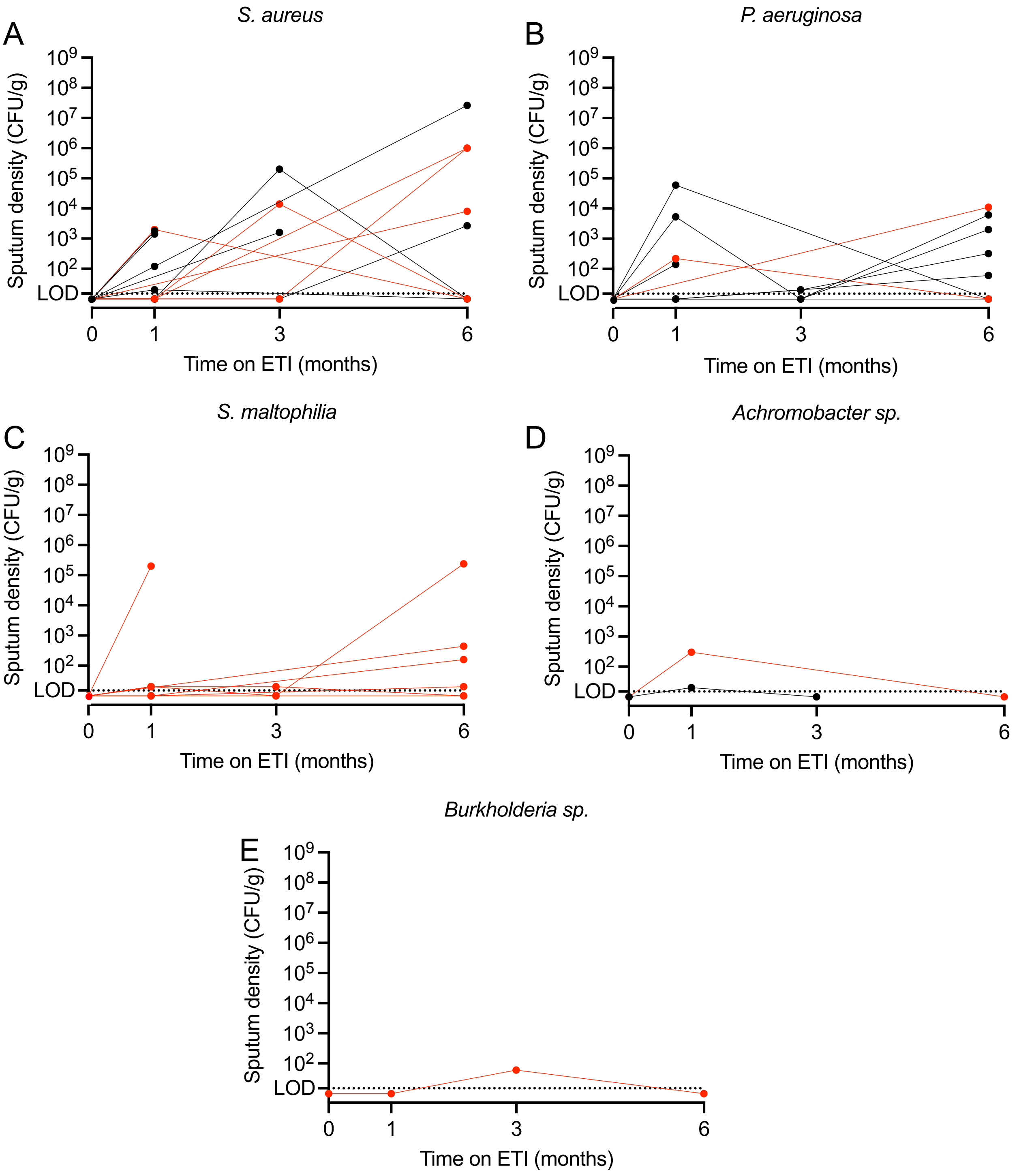

Figure S6. Some subjects become culture-positive for CF pathogens after starting ETI. Density of *S. aureus* (n=12) (A), *P. aeruginosa* (n=9) (B), *S. maltophilia* (n=9) (C), *Achromobacter spp*. (n=2) (D), and *Burkholderia spp*. (n=1) (E) measured by quantitative culture for participants who were culture-negative for the indicated pathogen at the baseline visit but became culture-positive after starting ETI. Participants represented with red lines had no positive cultures reported for the indicated organism for the two years prior to the baseline

visit according to CFF patient registry data (see methods). Participants with a registry record of at least one prior positive sputum culture for the indicated organism in the 2 years preceding the baseline visit, or participants whose registry records were unavailable are represented by black lines. The limit of detection is 20 cfu/ml and is indicated by a dotted line.

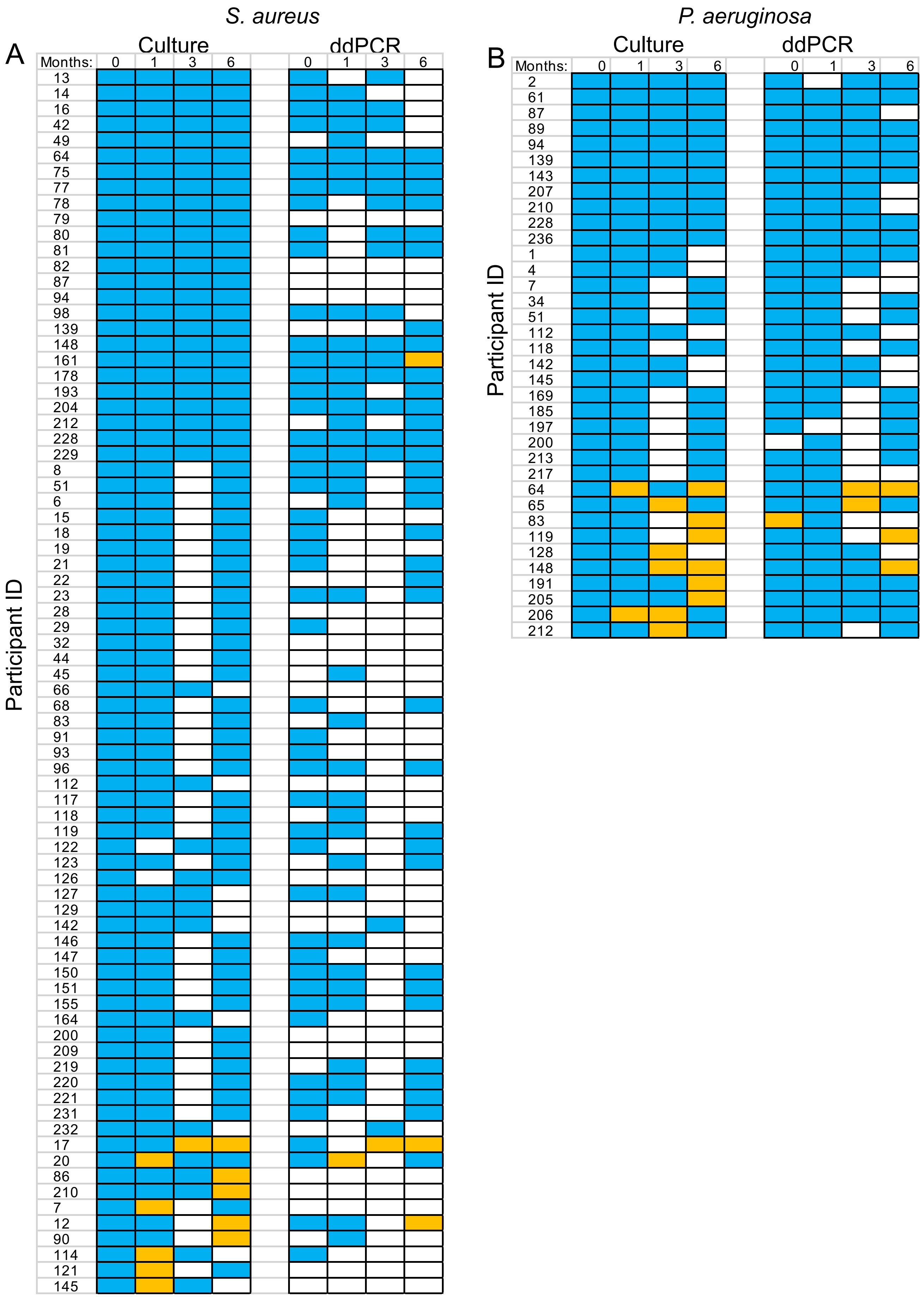
S. aureus
A
Culture
ddPCR
Months: 0 1 3 6
Participant ID
P. aeruginosa
B
Culture
ddPCR
Months: 0 1 3 6
Participant ID

Figure S7. Concordance between pathogen detection by culture and droplet digital PCR in participants culture-positive for *P. aeruginosa* and *S. aureus*.

Participants who were sputum culture positive for *S. aureus* (A) or *P. aeruginosa* (B) at baseline, provided at least two sputum samples post-ETI, and at least one post-baseline visit was culture positive are represented in rows in and detection of the indicated organisms by culture (left) and ddPCR (right) is shown for each study time point in columns. Positive culture or ddPCR results are indicated in blue; negative culture or ddPCR results are indicated in yellow; missing data is indicated by white. Participants that became repeatedly culture negative are shown in Fig 3.

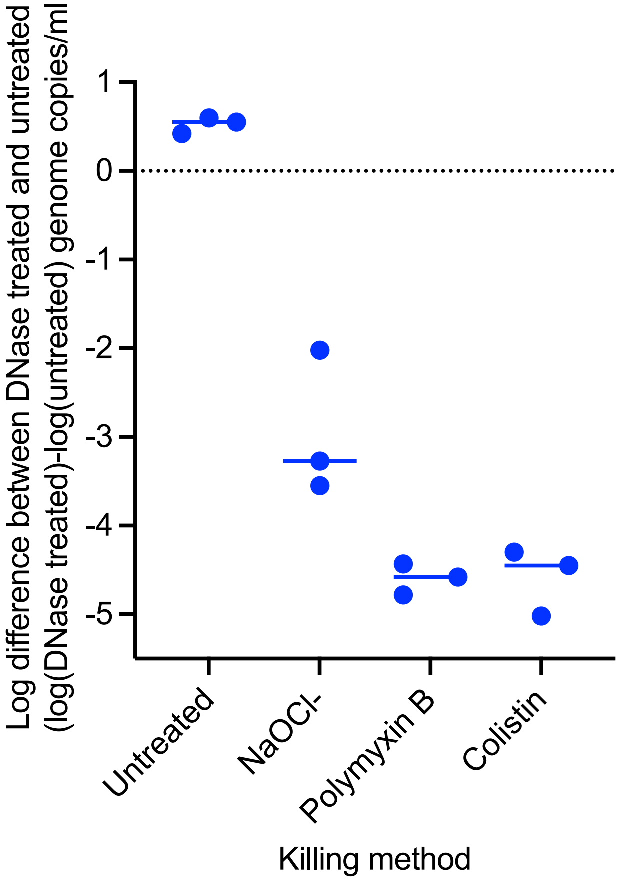

Figure S8. DNAse treatment markedly reduces DNA from dead cells. The log difference in genome copies/ml detected by ddPCR after DNAse treatment of *P. aeruginosa* cultures exposed to indicated bactericidal agents. Exponentially growing cells were completely killed by exposure to 8.06 mM NaOCl (to model killing by reactive oxygen species),or 50 ug/ml Polymyxin B or 50 ug/ml colistin (to model killing by antimicrobial peptides) for 2 hours. DNA was extracted either using the Qiagen microbiome kit which includes a DNase digestion prior to lysis of bacterial cells (DNase treated) or the Qiagen PowerSoil Pro kit which does not include a DNAse treatment (untreated). DNAse treatment reduced genome copies detected in cultures exposed to bactericidal agents (one way ANOVA; $p<0.0001$).

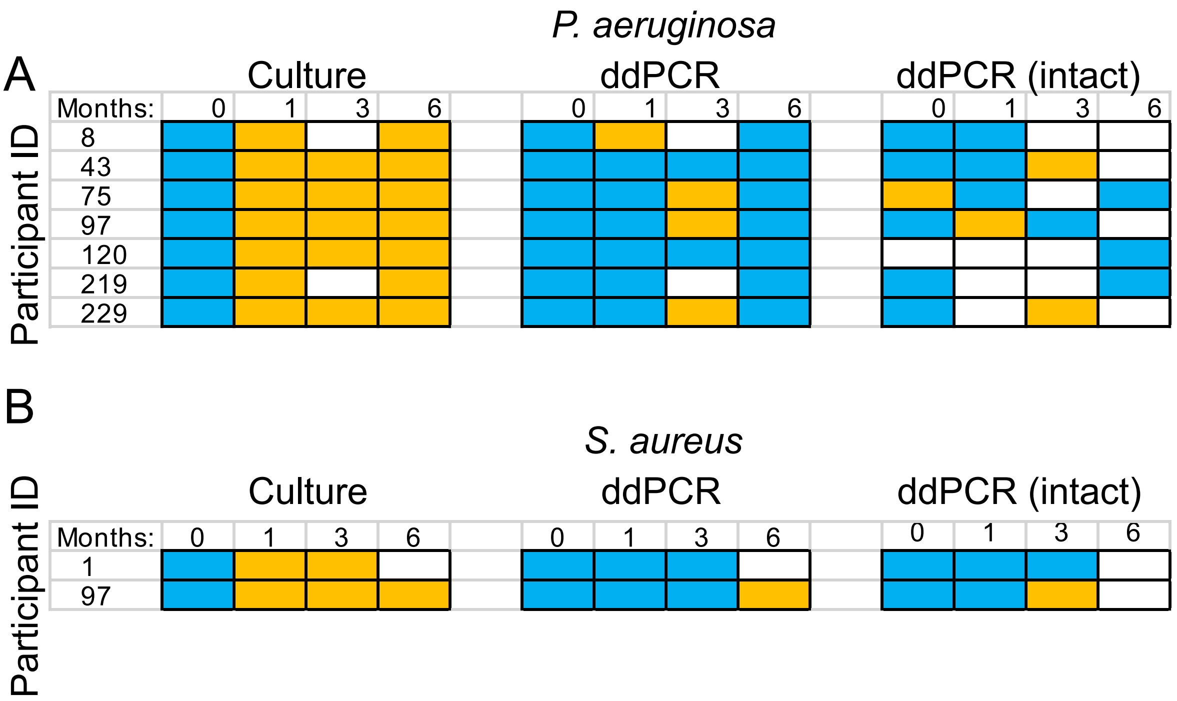

Figure S9. Intact *P. aeruginosa* cells can be detected in sputum months after sputum becomes *P. aeruginosa* culture negative. Participants who were become repeatedly sputum culture negative for *P. aeruginosa* (A) or *S. aureus* (B) after the baseline visit are indicated in rows. Detection of *P. aeruginosa* by culture (left panel, labeled as "Culture"); ddPCR of untreated sputum (middle panel, labeled as "ddPCR") or sputum treated with an extracellular DNase prior to DNA isolation (right panel, labeled as "ddPCR (intact)") for each study time point are shown in columns. Positive results are indicated in blue; results are indicated in yellow; missing data is indicated by white.

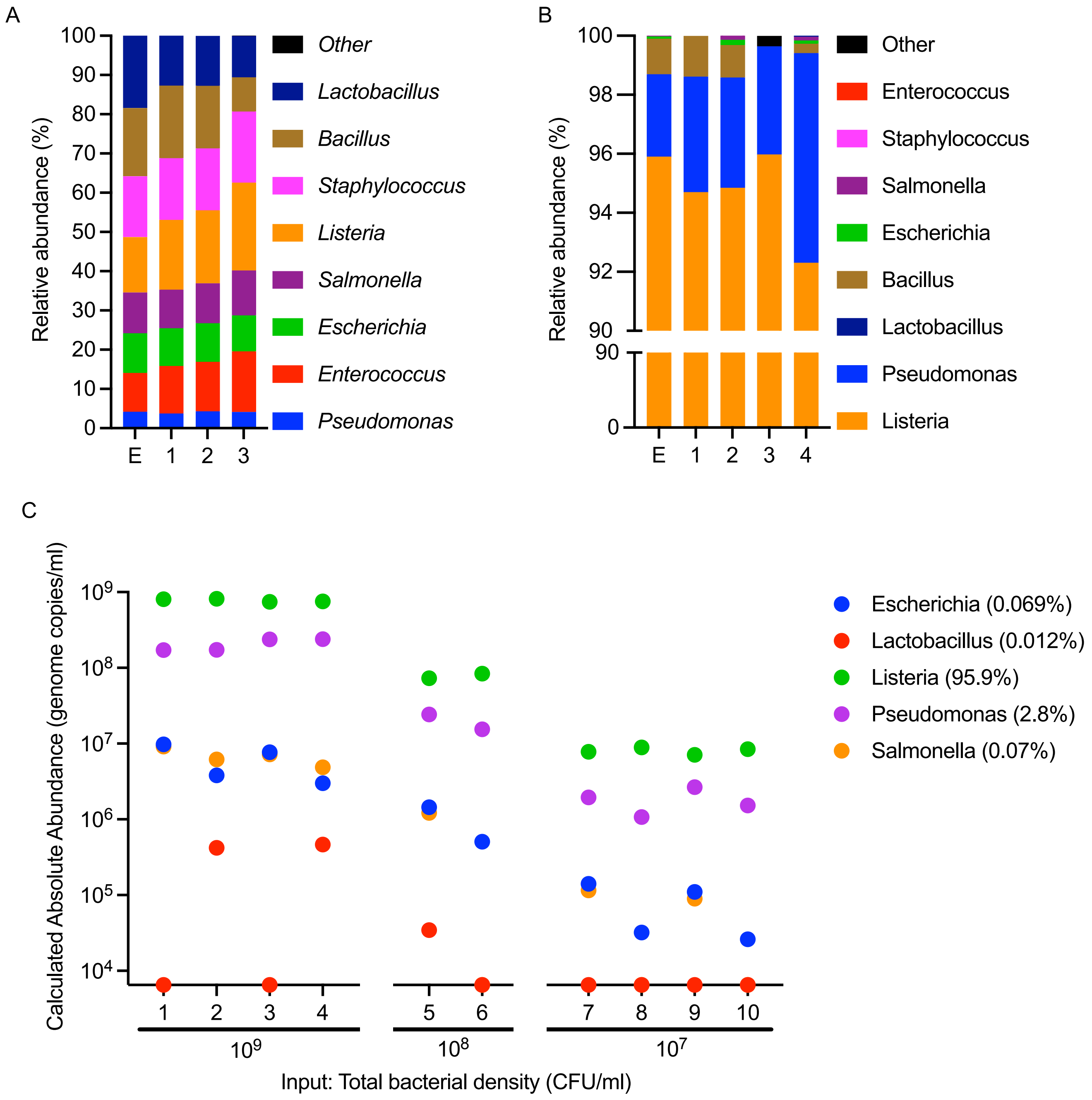

Figure S10. Validation of 16S analysis.

A&B. Relative abundance of ZymoBIOMICS microbial community standards to validate 16S analysis performed in this study. "E" indicates expected results, and data labeled "1-4" represent replicate aliquots of the microbial community standards that were separately DNA-extracted and sequenced. ZymoBIOMICS

community standard representing a relatively even distribution of genera (A) and ZymoBIOMICS II community standard representing a logarithmic distribution of genera (B) were both tested.

C. Calculated absolute abundance for replicates of serial dilutions of the ZymoBIOMICS II determined by multiplying the total 16S concentration as measured by 16S sequencing by the relative abundance in B.

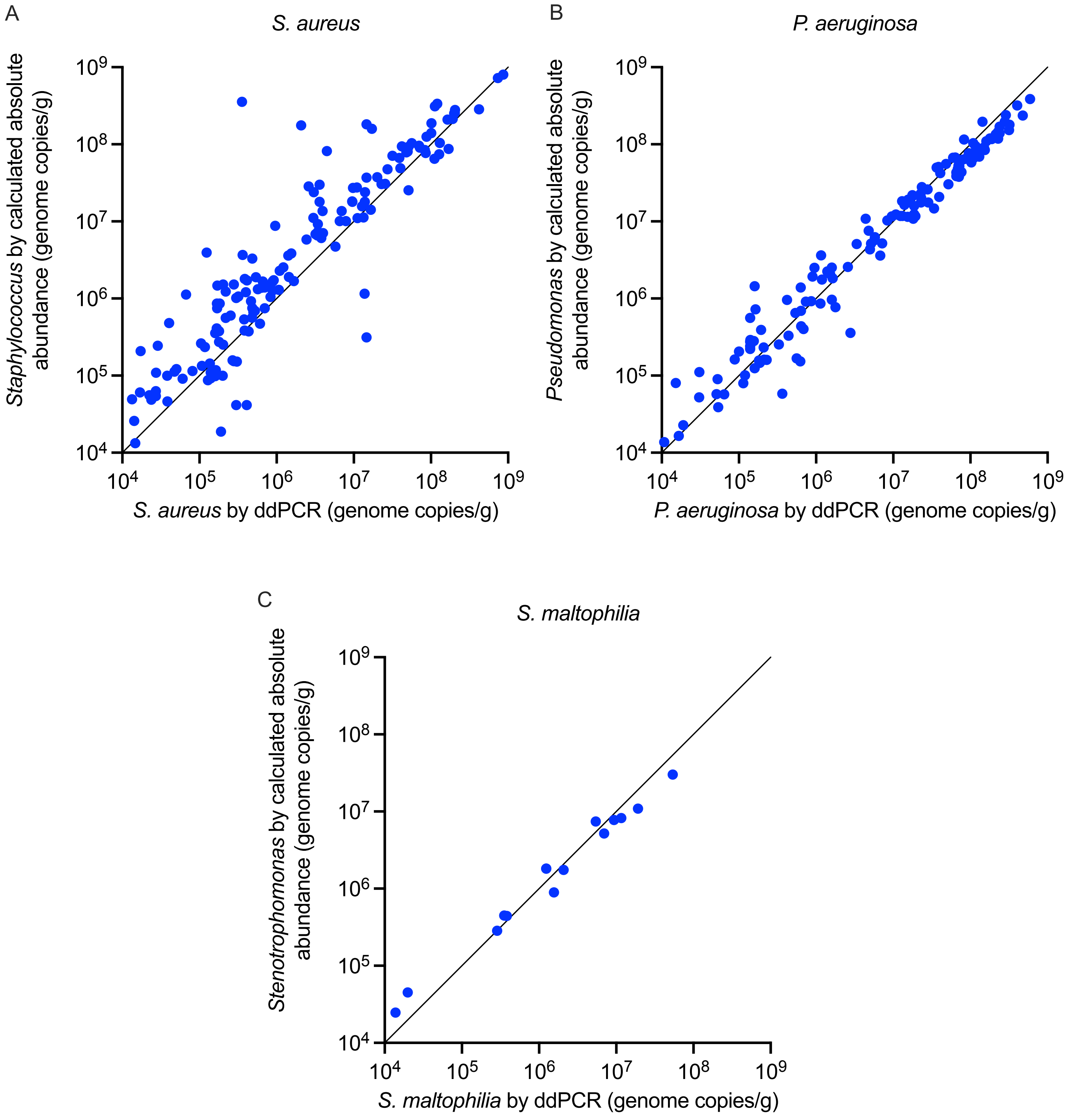

Figure S11. Validation of calculated absolute abundance data. Relationship between the absolute abundance of *S. aureus* (A). *P. aeruginosa* (B), *S. maltophilia* (C) calculated by multiplying the 16S rRNA abundance (as measured by ddPCR) and genus relative abundance (called “calculated absolute abundance”) (Y

axis), and the absolute abundance of the corresponding CF pathogen species measured by species-specific ddPCR (X-axis). Data is shown for samples with calculated absolute abundance values higher than 5000 genome copies/ml for each organism (the limited of detection for calculated absolute abundance values). Each point represents a study sample (from any time point). The black line marks a 1:1 correlation between calculated absolute abundance and ddPCR values. Staphylococcus genomes copies /ml measured by the calculated absolute abundance method are likely higher than *S. aureus* values measured by species-specific ddPCR because other staphylococcus spp such as *S. epidermidus* can be present in sputum. Linear regression of log transformed *S. aureus* values (A) yields a slope of 0.7979 (95% CI: 0.7414 - 0.8544) and r^2 of 0.8206; *P. aeruginosa* (B) yields a slope 0.8027 (95% CI: 0.7596 – 0.8457) and r^2 value of 0.9061; *S. maltophilia* (c) yields a slope of 0.6915 (95% CI: 0.5564 – 0.8183) and r^2 value of 0.9072.

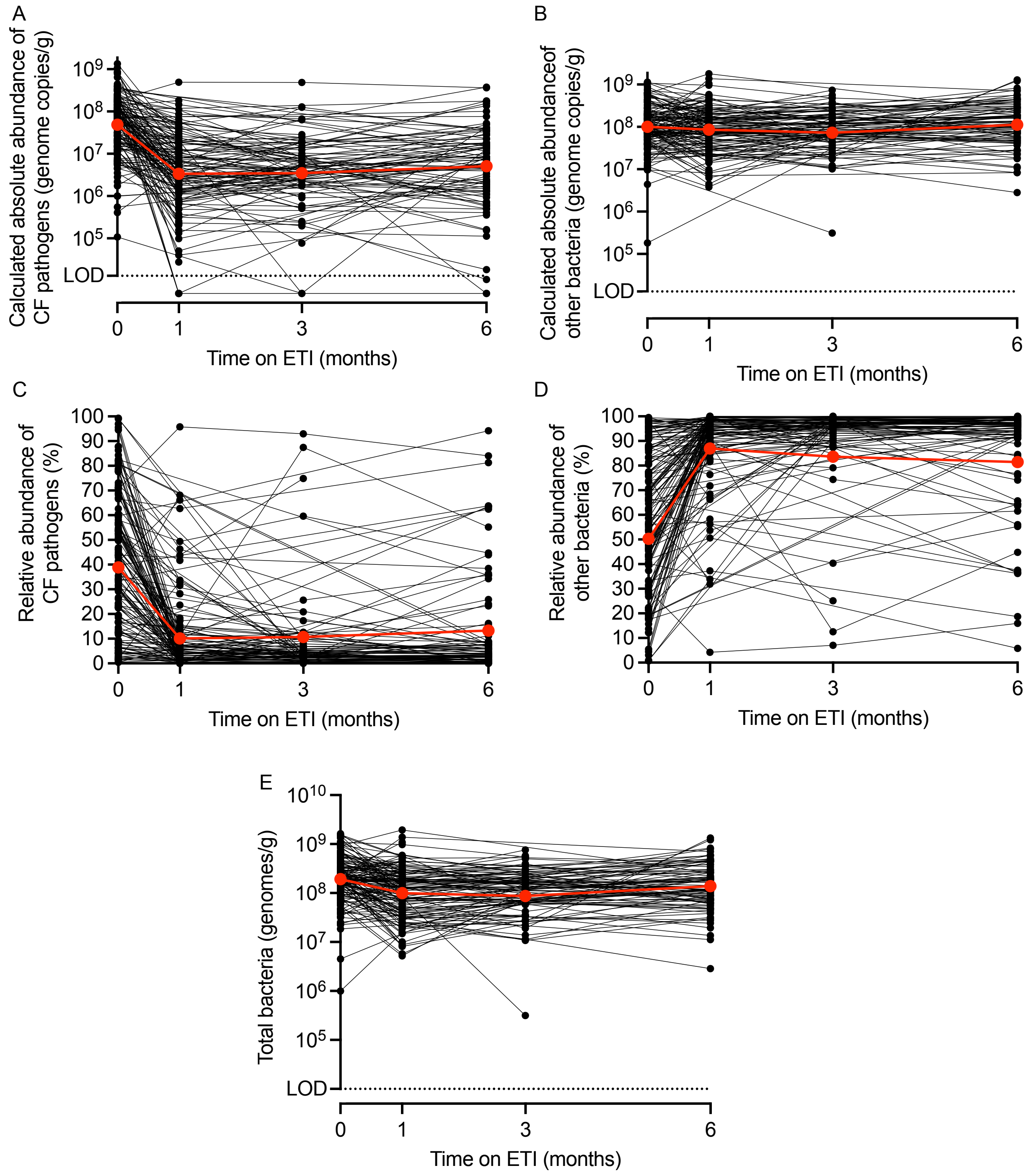

Figure S12. Change in abundance of traditional CF pathogen and other genera as determined by sequencing.

A&B. Change in calculated absolute abundance of traditional CF pathogens (A) and other genera (B) for 145 subjects for which sufficient sputum was available at visit 1 and at least one post-ETI visit. “Traditional CF pathogen” genera include *Achromobacter, Burkholderia, Corynebacterium, Escherichia/Shigella, Haemophilus, Inquilinus, Pandoraea, Pseudomonas, Staphylococcus, Stenotrophomonas* and the sum of these genera are represented in (A). "Other organisms"

genera include *Streptococcus, Prevotella, Veillonella, Rothia*, and all others, the sum of these genera are represented in (B).

C&D. Change in relative abundance of traditional CF pathogens (A) and other genera (B) in the sputum samples shown in A&B.

E. Change in total bacterial abundance as measured by16S ddPCR. Black lines represent individual participants: red lines indicate the geometric mean of the data.

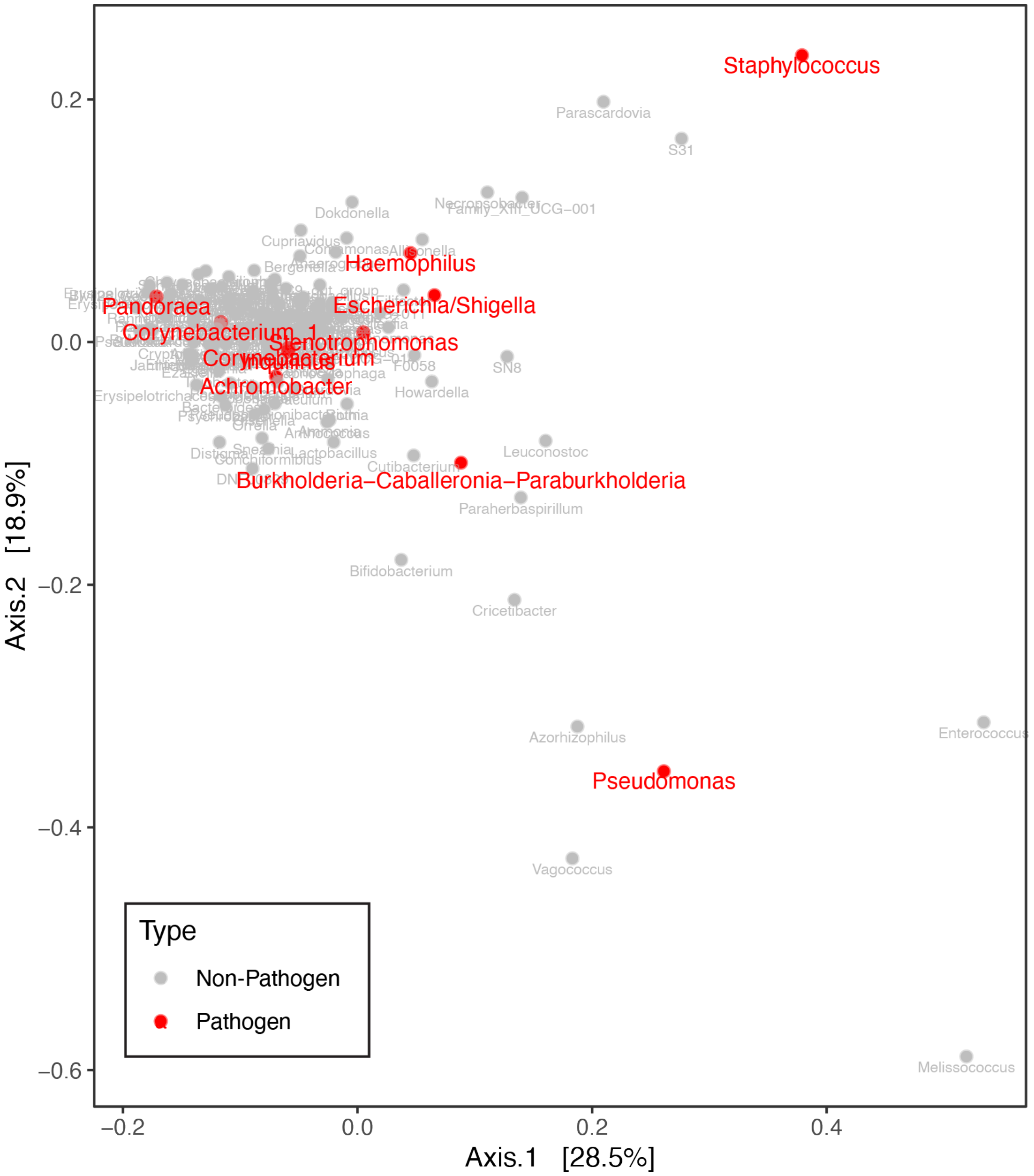

Figure S13. Taxon contributions to the Multidimensional Scaling (MDS) ordination of the samples. Taxon contributions refer to the Bray-Curtis index measurements of pair-wise dissimilarity performed on all reads from each subject shown in Figure 7A. Red symbols and labels indicate traditional CF pathogens, grey symbols and labels indicate other organisms (see key).

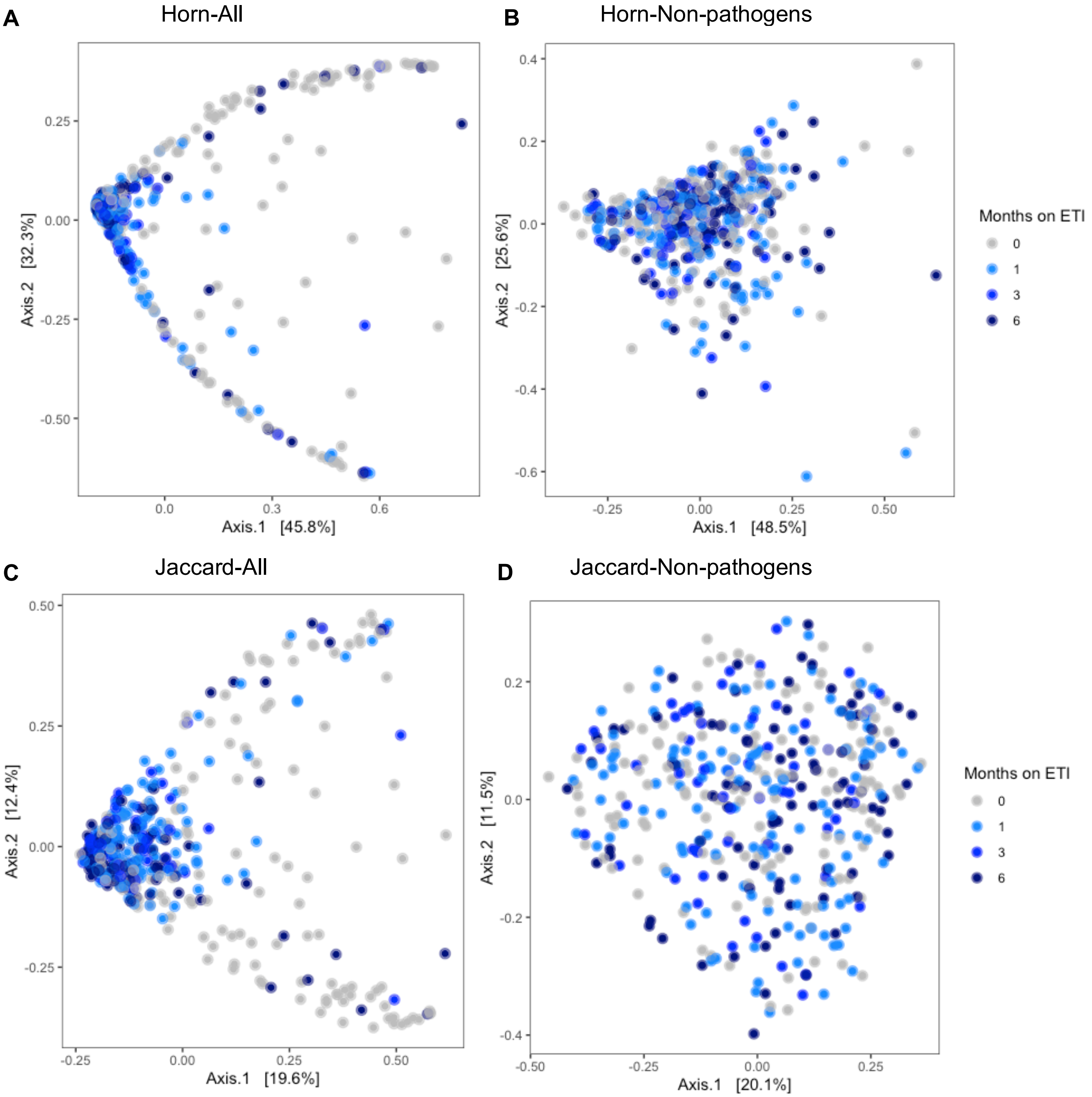

Figure S14. Changes in sputum microbial composition after ETI are driven by decreases in traditional CF pathogens. Multidimensional scaling (MDS) representation of the Horn (A&B) and Jaccard (B&C) pair-wise dissimilarity values of genera relative abundances measurements in each sputum sample to all other samples. **A and C.** Horn (A) and Jaccard (C) pair-wise dissimilarity values were calculated using all read counts for each taxon from each subject. **B and D.** To examine the effect of CF pathogen abundance changes on observed shifts in sputum microbial

composition, sequencing reads from traditional CF pathogen genera were computationally removed (see text) and Horn (B) and Jaccard (B) pair-wise dissimilarity values were re-calculated. Grey indicates samples collected pre-ETI, light blue 1 month post-ETI, blue 3 months post-ETI, and dark blue 6 months post-ETI.

Figure S15. Enrollment diagram

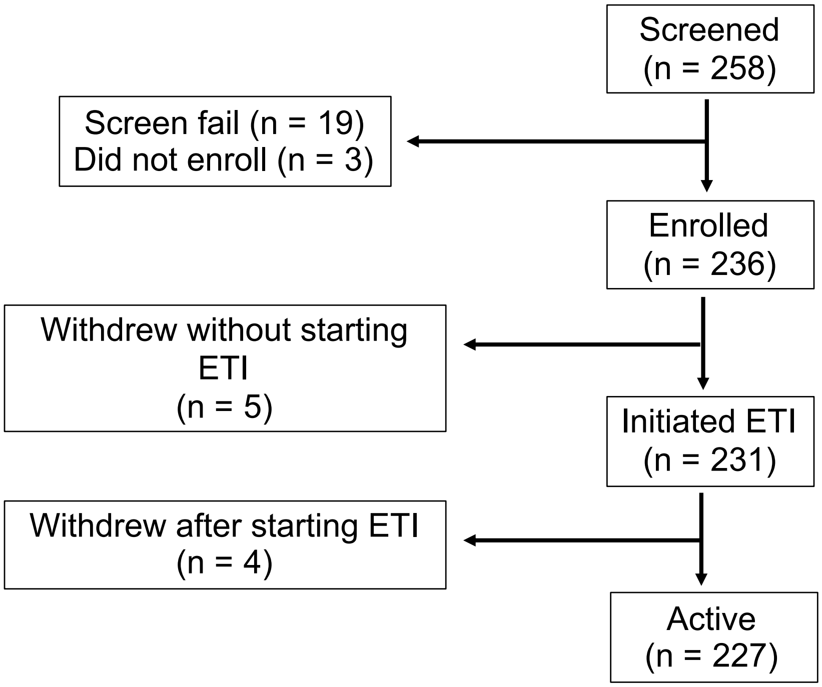

Supplement: Supplemental data [file jci-133-167957-s101.pdf]
